# A saturated map of common genetic variants associated with human height

**DOI:** 10.1038/s41586-022-05275-y

**Published:** 2022-10-12

**Authors:** Loïc Yengo, Sailaja Vedantam, Eirini Marouli, Julia Sidorenko, Eric Bartell, Saori Sakaue, Marielisa Graff, Anders U. Eliasen, Yunxuan Jiang, Sridharan Raghavan, Jenkai Miao, Joshua D. Arias, Sarah E. Graham, Ronen E. Mukamel, Cassandra N. Spracklen, Xianyong Yin, Shyh-Huei Chen, Teresa Ferreira, Heather H. Highland, Yingjie Ji, Tugce Karaderi, Kuang Lin, Kreete Lüll, Deborah E. Malden, Carolina Medina-Gomez, Moara Machado, Amy Moore, Sina Rüeger, Xueling Sim, Scott Vrieze, Tarunveer S. Ahluwalia, Masato Akiyama, Matthew A. Allison, Marcus Alvarez, Mette K. Andersen, Alireza Ani, Vivek Appadurai, Liubov Arbeeva, Seema Bhaskar, Lawrence F. Bielak, Sailalitha Bollepalli, Lori L. Bonnycastle, Jette Bork-Jensen, Jonathan P. Bradfield, Yuki Bradford, Peter S. Braund, Jennifer A. Brody, Kristoffer S. Burgdorf, Brian E. Cade, Hui Cai, Qiuyin Cai, Archie Campbell, Marisa Cañadas-Garre, Eulalia Catamo, Jin-Fang Chai, Xiaoran Chai, Li-Ching Chang, Yi-Cheng Chang, Chien-Hsiun Chen, Alessandra Chesi, Seung Hoan Choi, Ren-Hua Chung, Massimiliano Cocca, Maria Pina Concas, Christian Couture, Gabriel Cuellar-Partida, Rebecca Danning, E. Warwick Daw, Frauke Degenhard, Graciela E. Delgado, Alessandro Delitala, Ayse Demirkan, Xuan Deng, Poornima Devineni, Alexander Dietl, Maria Dimitriou, Latchezar Dimitrov, Rajkumar Dorajoo, Arif B. Ekici, Jorgen E. Engmann, Zammy Fairhurst-Hunter, Aliki-Eleni Farmaki, Jessica D. Faul, Juan-Carlos Fernandez-Lopez, Lukas Forer, Margherita Francescatto, Sandra Freitag-Wolf, Christian Fuchsberger, Tessel E. Galesloot, Yan Gao, Zishan Gao, Frank Geller, Olga Giannakopoulou, Franco Giulianini, Anette P. Gjesing, Anuj Goel, Scott D. Gordon, Mathias Gorski, Jakob Grove, Xiuqing Guo, Stefan Gustafsson, Jeffrey Haessler, Thomas F. Hansen, Aki S. Havulinna, Simon J. Haworth, Jing He, Nancy Heard-Costa, Prashantha Hebbar, George Hindy, Yuk-Lam A. Ho, Edith Hofer, Elizabeth Holliday, Katrin Horn, Whitney E. Hornsby, Jouke-Jan Hottenga, Hongyan Huang, Jie Huang, Alicia Huerta-Chagoya, Jennifer E. Huffman, Yi-Jen Hung, Shaofeng Huo, Mi Yeong Hwang, Hiroyuki Iha, Daisuke D. Ikeda, Masato Isono, Anne U. Jackson, Susanne Jäger, Iris E. Jansen, Ingegerd Johansson, Jost B. Jonas, Anna Jonsson, Torben Jørgensen, Ioanna-Panagiota Kalafati, Masahiro Kanai, Stavroula Kanoni, Line L. Kårhus, Anuradhani Kasturiratne, Tomohiro Katsuya, Takahisa Kawaguchi, Rachel L. Kember, Katherine A. Kentistou, Han-Na Kim, Young Jin Kim, Marcus E. Kleber, Maria J. Knol, Azra Kurbasic, Marie Lauzon, Phuong Le, Rodney Lea, Jong-Young Lee, Hampton L. Leonard, Shengchao A. Li, Xiaohui Li, Xiaoyin Li, Jingjing Liang, Honghuang Lin, Shih-Yi Lin, Jun Liu, Xueping Liu, Ken Sin Lo, Jirong Long, Laura Lores-Motta, Jian’an Luan, Valeriya Lyssenko, Leo-Pekka Lyytikäinen, Anubha Mahajan, Vasiliki Mamakou, Massimo Mangino, Ani Manichaikul, Jonathan Marten, Manuel Mattheisen, Laven Mavarani, Aaron F. McDaid, Karina Meidtner, Tori L. Melendez, Josep M. Mercader, Yuri Milaneschi, Jason E. Miller, Iona Y. Millwood, Pashupati P. Mishra, Ruth E. Mitchell, Line T. Møllehave, Anna Morgan, Soeren Mucha, Matthias Munz, Masahiro Nakatochi, Christopher P. Nelson, Maria Nethander, Chu Won Nho, Aneta A. Nielsen, Ilja M. Nolte, Suraj S. Nongmaithem, Raymond Noordam, Ioanna Ntalla, Teresa Nutile, Anita Pandit, Paraskevi Christofidou, Katri Pärna, Marc Pauper, Eva R. B. Petersen, Liselotte V. Petersen, Niina Pitkänen, Ozren Polašek, Alaitz Poveda, Michael H. Preuss, Saiju Pyarajan, Laura M. Raffield, Hiromi Rakugi, Julia Ramirez, Asif Rasheed, Dennis Raven, Nigel W. Rayner, Carlos Riveros, Rebecca Rohde, Daniela Ruggiero, Sanni E. Ruotsalainen, Kathleen A. Ryan, Maria Sabater-Lleal, Richa Saxena, Markus Scholz, Anoop Sendamarai, Botong Shen, Jingchunzi Shi, Jae Hun Shin, Carlo Sidore, Colleen M. Sitlani, Roderick C. Slieker, Roelof A. J. Smit, Albert V. Smith, Jennifer A. Smith, Laura J. Smyth, Lorraine Southam, Valgerdur Steinthorsdottir, Liang Sun, Fumihiko Takeuchi, Divya Sri Priyanka Tallapragada, Kent D. Taylor, Bamidele O. Tayo, Catherine Tcheandjieu, Natalie Terzikhan, Paola Tesolin, Alexander Teumer, Elizabeth Theusch, Deborah J. Thompson, Gudmar Thorleifsson, Paul R. H. J. Timmers, Stella Trompet, Constance Turman, Simona Vaccargiu, Sander W. van der Laan, Peter J. van der Most, Jan B. van Klinken, Jessica van Setten, Shefali S. Verma, Niek Verweij, Yogasudha Veturi, Carol A. Wang, Chaolong Wang, Lihua Wang, Zhe Wang, Helen R. Warren, Wen Bin Wei, Ananda R. Wickremasinghe, Matthias Wielscher, Kerri L. Wiggins, Bendik S. Winsvold, Andrew Wong, Yang Wu, Matthias Wuttke, Rui Xia, Tian Xie, Ken Yamamoto, Jingyun Yang, Jie Yao, Hannah Young, Noha A. Yousri, Lei Yu, Lingyao Zeng, Weihua Zhang, Xinyuan Zhang, Jing-Hua Zhao, Wei Zhao, Wei Zhou, Martina E. Zimmermann, Magdalena Zoledziewska, Linda S. Adair, Hieab H. H. Adams, Carlos A. Aguilar-Salinas, Fahd Al-Mulla, Donna K. Arnett, Folkert W. Asselbergs, Bjørn Olav Åsvold, John Attia, Bernhard Banas, Stefania Bandinelli, David A. Bennett, Tobias Bergler, Dwaipayan Bharadwaj, Ginevra Biino, Hans Bisgaard, Eric Boerwinkle, Carsten A. Böger, Klaus Bønnelykke, Dorret I. Boomsma, Anders D. Børglum, Judith B. Borja, Claude Bouchard, Donald W. Bowden, Ivan Brandslund, Ben Brumpton, Julie E. Buring, Mark J. Caulfield, John C. Chambers, Giriraj R. Chandak, Stephen J. Chanock, Nish Chaturvedi, Yii-Der Ida Chen, Zhengming Chen, Ching-Yu Cheng, Ingrid E. Christophersen, Marina Ciullo, John W. Cole, Francis S. Collins, Richard S. Cooper, Miguel Cruz, Francesco Cucca, L. Adrienne Cupples, Michael J. Cutler, Scott M. Damrauer, Thomas M. Dantoft, Gert J. de Borst, Lisette C. P. G. M. de Groot, Philip L. De Jager, Dominique P. V. de Kleijn, H. Janaka de Silva, George V. Dedoussis, Anneke I. den Hollander, Shufa Du, Douglas F. Easton, Petra J. M. Elders, A. Heather Eliassen, Patrick T. Ellinor, Sölve Elmståhl, Jeanette Erdmann, Michele K. Evans, Diane Fatkin, Bjarke Feenstra, Mary F. Feitosa, Luigi Ferrucci, Ian Ford, Myriam Fornage, Andre Franke, Paul W. Franks, Barry I. Freedman, Paolo Gasparini, Christian Gieger, Giorgia Girotto, Michael E. Goddard, Yvonne M. Golightly, Clicerio Gonzalez-Villalpando, Penny Gordon-Larsen, Harald Grallert, Struan F. A. Grant, Niels Grarup, Lyn Griffiths, Vilmundur Gudnason, Christopher Haiman, Hakon Hakonarson, Torben Hansen, Catharina A. Hartman, Andrew T. Hattersley, Caroline Hayward, Susan R. Heckbert, Chew-Kiat Heng, Christian Hengstenberg, Alex W. Hewitt, Haretsugu Hishigaki, Carel B. Hoyng, Paul L. Huang, Wei Huang, Steven C. Hunt, Kristian Hveem, Elina Hyppönen, William G. Iacono, Sahoko Ichihara, M. Arfan Ikram, Carmen R. Isasi, Rebecca D. Jackson, Marjo-Riitta Jarvelin, Zi-Bing Jin, Karl-Heinz Jöckel, Peter K. Joshi, Pekka Jousilahti, J. Wouter Jukema, Mika Kähönen, Yoichiro Kamatani, Kui Dong Kang, Jaakko Kaprio, Sharon L. R. Kardia, Fredrik Karpe, Norihiro Kato, Frank Kee, Thorsten Kessler, Amit V. Khera, Chiea Chuen Khor, Lambertus A. L. M. Kiemeney, Bong-Jo Kim, Eung Kweon Kim, Hyung-Lae Kim, Paulus Kirchhof, Mika Kivimaki, Woon-Puay Koh, Heikki A. Koistinen, Genovefa D. Kolovou, Jaspal S. Kooner, Charles Kooperberg, Anna Köttgen, Peter Kovacs, Adriaan Kraaijeveld, Peter Kraft, Ronald M. Krauss, Meena Kumari, Zoltan Kutalik, Markku Laakso, Leslie A. Lange, Claudia Langenberg, Lenore J. Launer, Loic Le Marchand, Hyejin Lee, Nanette R. Lee, Terho Lehtimäki, Huaixing Li, Liming Li, Wolfgang Lieb, Xu Lin, Lars Lind, Allan Linneberg, Ching-Ti Liu, Jianjun Liu, Markus Loeffler, Barry London, Steven A. Lubitz, Stephen J. Lye, David A. Mackey, Reedik Mägi, Patrik K. E. Magnusson, Gregory M. Marcus, Pedro Marques Vidal, Nicholas G. Martin, Winfried März, Fumihiko Matsuda, Robert W. McGarrah, Matt McGue, Amy Jayne McKnight, Sarah E. Medland, Dan Mellström, Andres Metspalu, Braxton D. Mitchell, Paul Mitchell, Dennis O. Mook-Kanamori, Andrew D. Morris, Lorelei A. Mucci, Patricia B. Munroe, Mike A. Nalls, Saman Nazarian, Amanda E. Nelson, Matt J. Neville, Christopher Newton-Cheh, Christopher S. Nielsen, Markus M. Nöthen, Claes Ohlsson, Albertine J. Oldehinkel, Lorena Orozco, Katja Pahkala, Päivi Pajukanta, Colin N. A. Palmer, Esteban J. Parra, Cristian Pattaro, Oluf Pedersen, Craig E. Pennell, Brenda W. J. H. Penninx, Louis Perusse, Annette Peters, Patricia A. Peyser, David J. Porteous, Danielle Posthuma, Chris Power, Peter P. Pramstaller, Michael A. Province, Qibin Qi, Jia Qu, Daniel J. Rader, Olli T. Raitakari, Sarju Ralhan, Loukianos S. Rallidis, Dabeeru C. Rao, Susan Redline, Dermot F. Reilly, Alexander P. Reiner, Sang Youl Rhee, Paul M. Ridker, Michiel Rienstra, Samuli Ripatti, Marylyn D. Ritchie, Dan M. Roden, Frits R. Rosendaal, Jerome I. Rotter, Igor Rudan, Femke Rutters, Charumathi Sabanayagam, Danish Saleheen, Veikko Salomaa, Nilesh J. Samani, Dharambir K. Sanghera, Naveed Sattar, Börge Schmidt, Helena Schmidt, Reinhold Schmidt, Matthias B. Schulze, Heribert Schunkert, Laura J. Scott, Rodney J. Scott, Peter Sever, Eric J. Shiroma, M. Benjamin Shoemaker, Xiao-Ou Shu, Eleanor M. Simonsick, Mario Sims, Jai Rup Singh, Andrew B. Singleton, Moritz F. Sinner, J. Gustav Smith, Harold Snieder, Tim D. Spector, Meir J. Stampfer, Klaus J. Stark, David P. Strachan, Leen M. ‘t Hart, Yasuharu Tabara, Hua Tang, Jean-Claude Tardif, Thangavel A. Thanaraj, Nicholas J. Timpson, Anke Tönjes, Angelo Tremblay, Tiinamaija Tuomi, Jaakko Tuomilehto, Maria-Teresa Tusié-Luna, Andre G. Uitterlinden, Rob M. van Dam, Pim van der Harst, Nathalie Van der Velde, Cornelia M. van Duijn, Natasja M. van Schoor, Veronique Vitart, Uwe Völker, Peter Vollenweider, Henry Völzke, Niels H. Wacher-Rodarte, Mark Walker, Ya Xing Wang, Nicholas J. Wareham, Richard M. Watanabe, Hugh Watkins, David R. Weir, Thomas M. Werge, Elisabeth Widen, Lynne R. Wilkens, Gonneke Willemsen, Walter C. Willett, James F. Wilson, Tien-Yin Wong, Jeong-Taek Woo, Alan F. Wright, Jer-Yuarn Wu, Huichun Xu, Chittaranjan S. Yajnik, Mitsuhiro Yokota, Jian-Min Yuan, Eleftheria Zeggini, Babette S. Zemel, Wei Zheng, Xiaofeng Zhu, Joseph M. Zmuda, Alan B. Zonderman, John-Anker Zwart, 23andMe Research Team, Gabriel Cuellar Partida, VA Million Veteran Program, Yan Sun, DiscovEHR (DiscovEHR and MyCode Community Health Initiative), eMERGE (Electronic Medical Records and Genomics Network), Damien Croteau-Chonka, Lifelines Cohort Study, Judith M. Vonk, The PRACTICAL Consortium, Stephen Chanock, Loic Le Marchand, Understanding Society Scientific Group, Daniel I. Chasman, Yoon Shin Cho, Iris M. Heid, Mark I. McCarthy, Maggie C. Y. Ng, Christopher J. O’Donnell, Fernando Rivadeneira, Unnur Thorsteinsdottir, Yan V. Sun, E. Shyong Tai, Michael Boehnke, Panos Deloukas, Anne E. Justice, Cecilia M. Lindgren, Ruth J. F. Loos, Karen L. Mohlke, Kari E. North, Kari Stefansson, Robin G. Walters, Thomas W. Winkler, Kristin L. Young, Po-Ru Loh, Jian Yang, Tõnu Esko, Themistocles L. Assimes, Adam Auton, Goncalo R. Abecasis, Cristen J. Willer, Adam E. Locke, Sonja I. Berndt, Guillaume Lettre, Timothy M. Frayling, Yukinori Okada, Andrew R. Wood, Peter M. Visscher, Joel N. Hirschhorn

**Affiliations:** 1 Institute for Molecular Bioscience, The University of Queensland, Brisbane, Queensland Australia; 2 Division of Endocrinology, Boston Children’s Hospital, Boston, MA USA; 3 Program in Medical and Population Genetics, Broad Institute of MIT and Harvard, Cambridge, MA USA; 4 William Harvey Research Institute, Barts and the London School of Medicine and Dentistry, Queen Mary University of London, London, UK; 5 Harvard Medical School, Boston, MA USA; 6 Laboratory for Statistical Analysis, RIKEN Center for Integrative Medical Sciences, Yokohama, Japan; 7 Department of Statistical Genetics, Osaka University Graduate School of Medicine, Osaka, Japan; 8 Divisions of Genetics and Rheumatology, Brigham and Women’s Hospital and Department of Medicine, Harvard Medical School, Boston, MA USA; 9 Department of Epidemiology, Gillings School of Global Public Health, University of North Carolina at Chapel Hill, Chapel Hill, NC USA; 10 COPSAC, Copenhagen Prospective Studies on Asthma in Childhood, Herlev and Gentofte Hospital, University of Copenhagen, Copenhagen, Denmark; 11 Section for Bioinformatics, Department of Health Technology, Technical University of Denmark, Copenhagen, Denmark; 12 23andMe, Sunnyvale, CA USA; 13 Department of Veterans Affairs, Eastern Colorado Healthcare System, Aurora, CO USA; 14 Division of Biomedical Informatics and Personalized Medicine, University of Colorado Anschutz Medical Campus, Aurora, CO USA; 15 Division of Cancer Epidemiology and Genetics, National Cancer Institute, Rockville, MD USA; 16 Department of Internal Medicine, Division of Cardiology, University of Michigan, Ann Arbor, MI USA; 17 Division of Genetics, Department of Medicine, Brigham and Women’s Hospital, Boston, MA USA; 18 Department of Medicine, Harvard Medical School, Boston, MA USA; 19 Department of Genetics, University of North Carolina at Chapel Hill, Chapel Hill, NC USA; 20 Department of Biostatistics and Epidemiology, School of Public Health and Health Sciences, University of Massachusetts, Amherst, MA USA; 21 Department of Biostatistics and Center for Statistical Genetics, University of Michigan School of Public Health, Ann Arbor, MI USA; 22 Department of Biostatistics and Data Science, Wake Forest School of Medicine, Winston-Salem, NC USA; 23 Big Data Institute, Li Ka Shing Centre for Health Information and Discovery, University of Oxford, Oxford, UK; 24 Genetics of Complex Traits, College of Medicine and Health, University of Exeter, Exeter, UK; 25 Center for Health Data Science, Faculty of Health and Medical Sciences, University of Copenhagen, Copenhagen, Denmark; 26 Wellcome Centre for Human Genetics, Nuffield Department of Medicine, University of Oxford, Oxford, UK; 27 Nuffield Department of Population Health, University of Oxford, Oxford, UK; 28 Institute of Genomics, Estonian Genome Centre, University of Tartu, Tartu, Estonia; 29 Department of Internal Medicine, Erasmus MC, University Medical Center Rotterdam, Rotterdam, The Netherlands; 30 Division of Biostatistics and Epidemiology, RTI International, Durham, NC USA; 31 Center for Primary Care and Public Health, University of Lausanne, Lausanne, Switzerland; 32 Swiss Institute of Bioinformatics, Lausanne, Switzerland; 33 Saw Swee Hock School of Public Health, National University of Singapore and National University Health System, Singapore, Singapore; 34 Department of Psychology, University of Minnesota, Minneapolis, MN USA; 35 Steno Diabetes Center Copenhagen, Herlev, Denmark; 36 Department of Biology, The Bioinformatics Center, University of Copenhagen, Copenhagen, Denmark; 37 Department of Ophthalmology, Graduate School of Medical Sciences, Kyushu University, Fukuoka, Japan; 38 Department of Family Medicine, University of California, San Diego, La Jolla, CA USA; 39 Department of Human Genetics, David Geffen School of Medicine at UCLA, Los Angeles, CA USA; 40 Novo Nordisk Foundation Center for Basic Metabolic Research, Faculty of Health and Medical Sciences, University of Copenhagen, Copenhagen, Denmark; 41 Department of Epidemiology, University of Groningen, University Medical Center Groningen, Groningen, The Netherlands; 42 Department of Bioinformatics, Isfahan University of Medical Sciences, Isfahan, Iran; 43 Institute of Biological Psychiatry, Mental Health Services, Copenhagen University Hospital, Copenhagen, Denmark; 44 Thurston Arthritis Research Center, University of North Carolina at Chapel Hill, Chapel Hill, NC USA; 45 Genomic Research on Complex diseases (GRC-Group), CSIR-Centre for Cellular and Molecular Biology, Hyderabad, India; 46 Department of Epidemiology, University of Michigan School of Public Health, Ann Arbor, MI USA; 47 Institute for Molecular Medicine Finland (FIMM), HiLIFE, University of Helsinki, Helsinki, Finland; 48 Molecular Genetics Section, Center for Precision Health Research, National Human Genome Research Institute, National Institutes of Health, Bethesda, MD USA; 49 Center for Applied Genomics, Children’s Hospital of Philadelphia, Philadelphia, PA USA; 50 Quantinuum Research, Wayne, PA USA; 51 Department of Genetics, University of Pennsylvania, Philadelphia, PA USA; 52 Department of Cardiovascular Sciences, University of Leicester, Leicester, UK; 53 NIHR Leicester Biomedical Research Centre, Glenfield Hospital, Leicester, UK; 54 Cardiovascular Health Research Unit, Department of Medicine, University of Washington, Seattle, WA USA; 55 Department of Clinical Immunology, Copenhagen University Hospital, Rigshospitalet, Copenhagen, Denmark; 56 NovoNordic Center for Protein Research, Copenhagen University, Copenhagen, Denmark; 57 Department of Medicine, Brigham and Women’s Hospital, Boston, MA USA; 58 Division of Epidemiology, Department of Medicine, Vanderbilt University Medical Center, Nashville, TN USA; 59 Centre for Genomic and Experimental Medicine, Institute of Genetics and Cancer, University of Edinburgh, Edinburgh, UK; 60 Centre for Public Health, Queen’s University of Belfast, Belfast, UK; 61 Institute for Maternal and Child Health – IRCCS, Burlo Garofolo, Trieste, Italy; 62 Ocular Epidemiology, Singapore Eye Research Institute, Singapore National Eye Centre, Singapore, Singapore; 63 Department of Ophthalmology, National University of Singapore and National University Health System, Singapore, Singapore; 64 Institute of Biomedical Sciences, Academia Sinica, Taipei, Taiwan; 65 Graduate Institute of Medical Genomics and Proteomics, Medical College, National Taiwan University, Taipei, Taiwan; 66 Department of Internal Medicine, National Taiwan University Hospital, Taipei, Taiwan; 67 Department of Pathology and Laboratory Medicine, University of Pennsylvania, Philadelphia, PA USA; 68 Center for Spatial and Functional Genomics, Division of Human Genetics, Children’s Hospital of Philadelphia, Philadelphia, PA USA; 69 Cardiovascular Disease Initiative, Broad Institute of MIT and Harvard, Cambridge, MA USA; 70 Institute of Population Health Sciences, National Health Research Institutes, Zhunan, Taiwan; 71 Department of Kinesiology, Faculty of Medicine, Université Laval, Québec City, Quebec Canada; 72 Diamantina Institute, The University of Queensland, Brisbane, Queensland Australia; 73 Division of Preventive Medicine, Brigham and Women’s Hospital, Boston, MA USA; 74 Division of Statistical Genomics, Department of Genetics, Washington University School of Medicine, St Louis, MO USA; 75 Institute of Clinical Molecular Biology, Christian-Albrechts University of Kiel, Kiel, Germany; 76 Vth Department of Medicine, Medical Faculty Mannheim, Heidelberg University, Mannheim, Germany; 77 Dipartimento di Scienze Mediche Chirurgiche e Sperimentali, Università degli Studi di Sassari, Sassari, Italy; 78 Department of Epidemiology, Erasmus MC, University Medical Center Rotterdam, Rotterdam, The Netherlands; 79 Section of Statistical Multi-omics, Department of Clinical and Experimental Medicine, University of Surrey, Guildford, UK; 80 Department of Biostatistics, Boston University School of Public Health, Boston, MA USA; 81 Center for Data and Computational Sciences, VA Boston Healthcare System, Boston, MA USA; 82 Department of Genetic Epidemiology, University of Regensburg, Regensburg, Germany; 83 Department of Internal Medicine II, University Hospital Regensburg, Regensburg, Germany; 84 Department of Nutrition and Dietetics, School of Health and Education, Harokopio University of Athens, Athens, Greece; 85 Center for Precision Medicine, Wake Forest School of Medicine, Medical Center Boulevard, Winston-Salem, NC USA; 86 Genome Institute of Singapore, Agency for Science, Technology and Research, Singapore, Singapore; 87 Health Services and Systems Research, Duke-NUS Medical School, Singapore, Singapore; 88 Institute of Human Genetics, Universitätsklinikum Erlangen, Friedrich-Alexander-Universität Erlangen-Nürnberg, Erlangen, Germany; 89 Institute of Cardiovascular Science, Faculty of Population Health, University College London, London, UK; 90 Survey Research Center, Institute for Social Research, University of Michigan, Ann Arbor, MI USA; 91 Computational Genomics Department, National Institute of Genomic Medicine, Mexico City, Mexico; 92 Institute of Genetic Epidemiology, Medical University of Innsbruck, Innsbruck, Austria; 93 Department of Medicine, Surgery and Health Sciences, University of Trieste, Trieste, Italy; 94 Institute of Medical Informatics and Statistics, Kiel University, Kiel, Germany; 95 Eurac Research, Institute for Biomedicine, Affiliated Institute of the University of Lübeck, Bolzano, Italy; 96 Radboud University Medical Center, Radboud Institute for Health Sciences, Department for Health Evidence, Nijmegen, The Netherlands; 97 Jackson Heart Study, Department of Medicine, University of Mississippi, Jackson, MS USA; 98 Nanjing University of Chinese Medicine, Nanjing, China; 99 Research Unit of Molecular Epidemiology, Institute of Epidemiology, Helmholtz Zentrum München Research Center for Environmental Health, Neuherberg, Germany; 100 Institute of Epidemiology, Helmholtz Zentrum München Research Center for Environmental Health, Neuherberg, Germany; 101 Department of Epidemiology Research, Statens Serum Institut, Copenhagen, Denmark; 102 Cardiovascular Medicine, Radcliffe Department of Medicine, University of Oxford, John Radcliffe Hospital, Oxford, UK; 103 Genetic Epidemiology, QIMR Berghofer Medical Research Institute, Brisbane, Queensland Australia; 104 Department of Biomedicine (Human Genetics) and iSEQ Center, Aarhus University, Aarhus, Denmark; 105 The Lundbeck Foundation Initiative for Integrative Psychiatric Research, iPSYCH, Aarhus, Denmark; 106 BiRC—Bioinformatics Research Centre, Aarhus University, Aarhus, Denmark; 107 The Institute for Translational Genomics and Population Sciences, Department of Pediatrics, The Lundquist Institute for Biomedical Innovation at Harbor-UCLA Medical Center, Torrance, CA USA; 108 Department of Medical Sciences, Uppsala University, Uppsala, Sweden; 109 Division of Public Health Sciences, Fred Hutchinson Cancer Research Center, Seattle, WA USA; 110 Danish Headache Center, Department of Neurology, Copenhagen University Hospital, Rigshospitalet, Rigshospitalet, Copenhagen Denmark; 111 Department of Public Health and Welfare, Finnish Institute for Health and Welfare, Helsinki, Finland; 112 MRC Integrative Epidemiology Unit, University of Bristol, Bristol, UK; 113 Bristol Dental School, University of Bristol, Bristol, UK; 114 Department of Neurology, Boston University School of Medicine, Boston, MA USA; 115 Framingham Heart Study, Framingham, MA USA; 116 Department of Genetics and Bioinformatics, Dasman Diabetes Institute, Kuwait City, Kuwait; 117 Department of Clinical Sciences in Malmö, Lund University, Malmö, Sweden; 118 Veterans Affairs Boston Healthcare System, Boston, MA USA; 119 Clinical Division of Neurogeriatrics, Department of Neurology, Medical University of Graz, Graz, Austria; 120 Institute for Medical Informatics, Statistics and Documentation, Medical University of Graz, Graz, Austria; 121 School of Medicine and Public Health, University of Newcastle, Callaghan, New South Wales Australia; 122 Institute for Medical Informatics, Statistics and Epidemiology, University of Leipzig, Medical Faculty, Leipzig, Germany; 123 LIFE Research Center for Civilization Diseases, University of Leipzig, Medical Faculty, Leipzig, Germany; 124 Department of Biological Psychology, Behaviour and Movement Sciences, Vrije Universiteit Amsterdam, Amsterdam, The Netherlands; 125 Department of Epidemiology, Harvard T.H. Chan School of Public Health, Boston, MA USA; 126 School of Public Health and Emergency Management, Southern University of Science and Technology, Shenzhen, China; 127 Institute for Global Health and Development, Peking University, Beijing, China; 128 Programs in Metabolism and Medical and Population Genetics, Broad Institute of Harvard and MIT, Cambridge, MA USA; 129 Departamento de Medicina Genómica y Toxicología Ambiental, Instituto de Investigaciones Biomédicas, Universidad Nacional Autónoma de México Ciudad Universitaria, Mexico City, Mexico; 130 Unidad de Biología Molecular y Medicina Genómica, Instituto Nacional de Ciencias Médicas y Nutrición, Mexico City, Mexico; 131 Division of Endocrine and Metabolism, Tri-Service General Hospital Songshan Branch, Taipei, Taiwan; 132 Shanghai Institute of Nutrition and Health, University of Chinese Academy of Sciences, Chinese Academy of Sciences, Shanghai, China; 133 Division of Genome Science, Department of Precision Medicine, National Institute of Health, Cheongju, Republic of Korea; 134 Biomedical Technology Research Center, Tokushima Research Institute, Otsuka Pharmaceutical Co., Tokushima, Japan; 135 Research Institute, National Center for Global Health and Medicine, Tokyo, Japan; 136 Department of Molecular Epidemiology, German Institute of Human Nutrition Potsdam-Rehbruecke, Nuthetal, Germany; 137 German Center for Diabetes Research (DZD), Neuherberg, Germany; 138 Department of Complex Trait Genetics, Center for Neurogenomics and Cognitive Research, Amsterdam Neuroscience, Vrije Universiteit Amsterdam, Amsterdam, The Netherlands; 139 Department of Child and Adolescent Psychiatry and Pediatric Psychology, Section Complex Trait Genetics, Amsterdam Neuroscience, Vrije Universiteit Medical Center, Amsterdam, The Netherlands; 140 Department of Biobank Research, Umeå University, Umeå, Sweden; 141 Department of Odontology, Umeå University, Umeå, Sweden; 142 Institute of Molecular and Clinical Ophthalmology Basel, Basel, Switzerland; 143 Privatpraxis Prof Jonas und Dr Panda-Jonas, Heidelberg, Germany; 144 Department of Ophthalmology, Medical Faculty Mannheim, Heidelberg University, Mannheim, Germany; 145 Beijing Institute of Ophthalmology, Beijing Tongren Eye Center, Beijing Tongren Hospital, Capital Medical University, Beijing Ophthalmology and Visual Sciences Key Laboratory, Beijing, China; 146 Center for Clinical Research and Prevention, Copenhagen University Hospital - Bispebjerg and Frederiksberg, Copenhagen, Denmark; 147 Department of Public Health, Faculty of Health and Medical Sciences, University of Copenhagen, Copenhagen, Denmark; 148 Faculty of Medicine, University of Kelaniya, Ragama, Sri Lanka; 149 Department of Geriatric and General Medicine, Osaka University Graduate School of Medicine, Suita, Japan; 150 Center for Genomic Medicine, Kyoto University Graduate School of Medicine, Kyoto, Japan; 151 Department of Psychiatry, University of Pennsylvania, Philadelphia, PA USA; 152 Centre for Global Health, Usher Institute, University of Edinburgh, Edinburgh, UK; 153 Centre for Cardiovascular Sciences, Queens Medical Research Institute, University of Edinburgh, Edinburgh, UK; 154 Medical Research Institute, Kangbuk Samsung Hospital, Sungkyunkwan University School of Medicine, Seoul, Republic of Korea; 155 Department of Clinical Research Design and Evaluation (SAIHST), Sungkyunkwan University, Seoul, Republic of Korea; 156 SYNLAB MVZ Humangenetik Mannheim, Mannheim, Germany; 157 Department of Clinical Sciences, Genetic and Molecular Epidemiology Unit, Lund University, Malmö, Sweden; 158 Department of Computer Science, University of Toronto, Toronto, Ontario Canada; 159 Department of Anthropology, University of Toronto at Mississauga, Mississauga, Ontario Canada; 160 Genomics Research Centre, Centre for Genomics and Personalised Health, School of Biomedical Sciences, Queensland University of Technology, Kelvin Grove, Queensland Australia; 161 Oneomics, Soonchunhyang Mirai Medical Center, Bucheon-si, Republic of Korea; 162 Laboratory of Neurogenetics, National Institute on Aging, National Institutes of Health, Bethesda, MD USA; 163 Center for Alzheimer’s and Related Dementias, National Institutes of Health, Bethesda, MD USA; 164 Data Tecnica International, Glen Echo, MD USA; 165 Cancer Genomics Research Laboratory, Leidos Biomedical Research, Rockville, MD USA; 166 Department of Population and Quantitative Health Sciences, Case Western Reserve University, Cleveland, OH USA; 167 Department of Medicine, University of Massachusetts Chan Medical School, Worcester, MA USA; 168 Center for Geriatrics and Gerontology, Taichung Veterans General Hospital, Taichung, Taiwan; 169 Montreal Heart Institute, Montreal, Quebec Canada; 170 Departments of Ophthalmology and Human Genetics, Radboud University Nijmegen Medical Center, Nijmegen, The Netherlands; 171 MRC Epidemiology Unit, University of Cambridge School of Clinical Medicine, Cambridge, UK; 172 Department of Clinical Science, Center for Diabetes Research, University of Bergen, Bergen, Norway; 173 Department of Clinical Sciences, Lund University Diabetes Centre, Malmö, Sweden; 174 Department of Clinical Chemistry, Fimlab Laboratories, Tampere, Finland; 175 Department of Clinical Chemistry, Finnish Cardiovascular Research Center - Tampere, Faculty of Medicine and Health Technology, Tampere University, Tampere, Finland; 176 Department of Cardiology, Heart Center, Tampere University Hospital, Tampere, Finland; 177 National and Kapodistrian University of Athens, Dromokaiteio Psychiatric Hospital, Athens, Greece; 178 Department of Twin Research and Genetic Epidemiology, King’s College London, London, UK; 179 NIHR Biomedical Research Centre at Guy’s and St Thomas’ Foundation Trust, London, UK; 180 Center for Public Health Genomics, University of Virginia School of Medicine, Charlottesville, VA USA; 181 MRC Human Genetics Unit, Institute of Genetics and Cancer, University of Edinburgh, Western General Hospital, Edinburgh, UK; 182 Department of Psychiatry and Department of Community Health and Epidemiology, Dalhousie University, Halifax, Nova Scotia Canada; 183 Institute of Psychiatric Phenomics and Genomics (IPPG), University Hospital, LMU Munich, Munich, Germany; 184 Institute for Medical Informatics, Biometry and Epidemiology, University Hospital Essen, Essen, Germany; 185 Diabetes Unit, Massachusetts General Hospital, Boston, MA USA; 186 Center for Genomic Medicine, Massachusetts General Hospital, Boston, MA USA; 187 Department of Psychiatry, Amsterdam Public Health and Amsterdam Neuroscience, Amsterdam UMC and Vrije Universiteit, Amsterdam, The Netherlands; 188 Biomedical and Translational Informatics Institute, Geisinger, Danville, PA USA; 189 Department of Genetics, Institute for Biomedical Informatics, Perelman School of Medicine, University of Pennsylvania, Philadelphia, PA USA; 190 MRC Population Health Research Unit, Nuffield Department of Population Health, University of Oxford, Oxford, UK; 191 Population Health Sciences, Bristol Medical School, University of Bristol, Bristol, UK; 192 Institute for Cardiogenetics, University of Lübeck, DZHK (German Research Centre for Cardiovascular Research) partner site Hamburg/Lübeck/Kiel and University Heart Center Lübeck, Lübeck, Germany; 193 Public Health Informatics Unit, Department of Integrated Health Sciences, Nagoya University Graduate School of Medicine, Nagoya, Japan; 194 Centre for Bone and Arthritis Research, Department of Internal Medicine and Clinical Nutrition, Institute of Medicine, Sahlgrenska Academy, University of Gothenburg, Gothenburg, Sweden; 195 Bioinformatics Core Facility, Sahlgrenska Academy, University of Gothenburg, Gothenburg, Sweden; 196 Korea Institute of Science and Technology, Gangneung Institute of Natural Products, Gangneung, Republic of Korea; 197 Department of Clinical Biochemistry, Lillebaelt Hospital, Kolding, Denmark; 198 Department of Human Genetics, Wellcome Sanger Institute, Hinxton, UK; 199 Department of Internal Medicine, Section of Gerontology and Geriatrics, Leiden University Medical Center, Leiden, The Netherlands; 200 Institute of Genetics and Biophysics A. Buzzati-Traverso, CNR, Naples, Italy; 201 Department of Clinical Biochemistry and Immunology, Hospital of Southern Jutland, Aabenraa, Denmark; 202 The National Centre for Register-based Research, University of Aarhus, Aarhus, Denmark; 203 Centre for Population Health Research, University of Turku and Turku University Hospital, Turku, Finland; 204 Research Centre of Applied and Preventive Cardiovascular Medicine, University of Turku, Turku, Finland; 205 Medical School, University of Split, Split, Croatia; 206 Algebra University College, Zagreb, Croatia; 207 The Charles Bronfman Institute for Personalized Medicine, Icahn School of Medicine at Mount Sinai, New York, NY USA; 208 Department of Environmental Medicine and Public Health, Icahn School of Medicine at Mount Sinai, New York, NY USA; 209 Aragon Institute of Engineering Research, University of Zaragoza, Zaragoza, Spain; 210 Centro de Investigación Biomédica en Red en Bioingeniería, Biomateriales y Nanomedicina (CIBER-BBN), Madrid, Spain; 211 Center for Non-Communicable Diseases, Karachi, Pakistan; 212 Department of Psychiatry, Interdisciplinary Center Psychopathology and Emotion Regulation, University of Groningen, University Medical Center Groningen, Groningen, The Netherlands; 213 Institute of Translational Genomics, Helmholtz Zentrum München, German Research Center for Environmental Health, Neuherberg, Germany; 214 Present Address: Oxford Centre for Diabetes, Endocrinology and Metabolism, Radcliffe Department of Medicine, University of Oxford, Churchill Hospital, Oxford, UK; 215 Hunter Medical Research Institute, New Lambton Heights, New South Wales Australia; 216 School of Medicine and Public Health, College of Health, Medicine and Wellbeing, The University of Newcastle, New Lambton Heights, New South Wales Australia; 217 IRCCS Neuromed, Pozzilli, Italy; 218 Department of Medicine, Division of Endocrinology, Diabetes and Nutrition, University of Maryland School of Medicine, Baltimore, MD USA; 219 Program for Personalized and Genomic Medicine, University of Maryland School of Medicine, Baltimore, MD USA; 220 Unit of Genomics of Complex Diseases, Sant Pau Biomedical Research Institute (IIB Sant Pau), Barcelona, Spain; 221 Cardiovascular Medicine Unit, Department of Medicine, Karolinska Institutet, Center for Molecular Medicine, Stockholm, Sweden; 222 Laboratory of Epidemiology and Population Sciences, National Institute on Aging, National Institutes of Health, Baltimore, MD USA; 223 Department of Biomedical Science, Hallym University, Chuncheon, Republic of Korea; 224 Istituto di Ricerca Genetica e Biomedica, Consiglio Nazionale delle Ricerche (CNR), Cagliari, Italy; 225 Department of Cell and Chemical Biology, Leiden University Medical Center, Leiden, The Netherlands; 226 Epidemiology and Data Science, Amsterdam UMC, location Vrije Universiteit Amsterdam, Amsterdam, The Netherlands; 227 Amsterdam Cardiovascular Sciences, Amsterdam, The Netherlands; 228 Department of Clinical Epidemiology, Leiden University Medical Center, Leiden, The Netherlands; 229 Icelandic Heart Association, Kópavogur, Iceland; 230 Wellcome Sanger Institute, Hinxton, UK; 231 deCODE Genetics/Amgen, Reykjavik, Iceland; 232 Mohn Nutrition Research Laboratory, Department of Clinical Science, University of Bergen, Bergen, Norway; 233 Department of Public Health Sciences, Parkinson School of Health Sciences and Public Health, Loyola University Chicago, Maywood, IL USA; 234 VA Palo Alto Health Care System, Palo Alto, CA USA; 235 Department of Medicine, Stanford University School of Medicine, Stanford, CA USA; 236 Institute for Community Medicine, University Medicine Greifswald, Greifswald, Germany; 237 DZHK (German Centre for Cardiovascular Research), partner site Greifswald, Greifswald, Germany; 238 Cardiology Division, Department of Pediatrics, University of California, San Francisco, Oakland, CA USA; 239 Centre for Cancer Genetic Epidemiology, University of Cambridge, Cambridge, UK; 240 Department of Public Health and Primary Care, University of Cambridge, Cambridge, UK; 241 Department of Cardiology, Leiden University Medical Center, Leiden, The Netherlands; 242 Central Diagnostics Laboratory, Division Laboratories, Pharmacy and Biomedical Genetics, University Medical Center Utrecht, Utrecht University, Utrecht, The Netherlands; 243 Department of Human Genetics, Leiden University Medical Center, Leiden, The Netherlands; 244 Laboratory Genetic Metabolic Diseases, Department of Clinical Chemistry, Amsterdam UMC, University of Amsterdam, Amsterdam, The Netherlands; 245 Core Facility Metabolomics, Amsterdam UMC, University of Amsterdam, Amsterdam, The Netherlands; 246 Department of Cardiology, Division Heart and Lungs, University Medical Center Utrecht, Utrecht University, Utrecht, The Netherlands; 247 Department of Cardiology, University of Groningen, University Medical Center Groningen, Groningen, The Netherlands; 248 Department of Epidemiology and Biostatistics, School of Public Health, Tongji Medical College, Huazhong University of Science and Technology, Wuhan, China; 249 NIHR Barts Cardiovascular Biomedical Research Centre, Barts and The London School of Medicine and Dentistry, Queen Mary University of London, London, UK; 250 Department of Ophthalmology, Beijing Tongren Hospital, Capital Medical University, Beijing, China; 251 Department of Epidemiology and Biostatistics, MRC-PHE Centre for Environment and Health, School of Public Health, Imperial College London, London, UK; 252 Department of Dermatology, Medical University of Vienna, Vienna, Austria; 253 Department of Research and Innovation, Division of Clinical Neuroscience, Oslo University Hospital, Oslo, Norway; 254 Department of Neurology, Oslo University Hospital, Oslo, Norway; 255 MRC Unit for Lifelong Health and Ageing at UCL, Institute of Cardiovascular Science, University College London, London, UK; 256 Institute of Genetic Epidemiology, Faculty of Medicine and Medical Center, University of Freiburg, Freiburg, Germany; 257 Department of Medicine IV – Nephrology and Primary Care, Faculty of Medicine and Medical Center, University of Freiburg, Freiburg, Germany; 258 Institute of Molecular Medicine, McGovern Medical School, University of Texas Health Science Center at Houston, Houston, TX USA; 259 Department of Medical Biochemistry, Kurume University School of Medicine, Kurume, Japan; 260 Rush Alzheimer’s Disease Center, Rush University Medical Center, Chicago, IL USA; 261 Department of Neurological Sciences, Rush University Medical Center, Chicago, IL USA; 262 Department of Genetic Medicine, Weill Cornell Medicine-Qatar, Doha, Qatar; 263 Department of Computer and Systems Engineering, Alexandria University, Alexandria, Egypt; 264 Department of Cardiology, German Heart Centre Munich, Technical University Munich, Munich, Germany; 265 Department of Cardiology, Ealing Hospital, London North West University Healthcare NHS Trust, London, UK; 266 Department of Epidemiology and Biostatistics, Imperial College London, London, UK; 267 Cardiovascular Epidemiology Unit, Department of Public Health and Primary Care, University of Cambridge, Strangeways Research Laboratory, Cambridge, UK; 268 Department of Computational Medicine and Bioinformatics, University of Michigan, Ann Arbor, MI USA; 269 Analytic and Translational Genetics Unit, Massachusetts General Hospital, Boston, MA USA; 270 Stanley Center for Psychiatric Research, Broad Institute of MIT and Harvard, Cambridge, MA USA; 271 Department of Nutrition, Gillings School of Global Public Health, University of North Carolina at Chapel Hill, Chapel Hill, NC USA; 272 Carolina Population Center, University of North Carolina at Chapel Hill, Chapel Hill, NC USA; 273 Department of Clinical Genetics, Erasmus MC, Rotterdam, The Netherlands; 274 Department of Radiology and Nuclear Medicine, Erasmus MC, Rotterdam, The Netherlands; 275 Latin American Brain Health (BrainLat), Universidad Adolfo Ibáñez, Santiago, Chile; 276 Unidad de Investigacion de Enfermedades Metabolicas and Direction of Nutrition, Instituto Nacional de Ciencias Medicas y Nutricion, Mexico City, Mexico; 277 Escuela de Medicina y Ciencias de la Salud, Tecnologico de Monterrey, Monterrey, Mexico; 278 Department of Epidemiology and Dean’s Office, College of Public Health, University of Kentucky, Lexington, KY USA; 279 Institute of Cardiovascular Science, Faculty of Population Health Sciences, University College London, London, UK; 280 Health Data Research UK and Institute of Health Informatics, University College London, London, UK; 281 KG Jebsen Center for Genetic Epidemiology, Department of Public Health and Nursing, Faculty of Medicine and Health Sciences, Norwegian University of Science and Technology, NTNU, Trondheim, Norway; 282 HUNT Research Centre, Department of Public Health and Nursing, Norwegian University of Science and Technology, Levanger, Norway; 283 Department of Endocrinology, Clinic of Medicine, St. Olavs Hospital, Trondheim University Hospital, Trondheim, Norway; 284 Department of Nephrology, University Hospital Regensburg, Regensburg, Germany; 285 Geriatric Unit, Azienda Toscana Centro, Florence, Italy; 286 Systems Genomics Laboratory, School of Biotechnology, Jawaharlal Nehru University (JNU), New Delhi, India; 287 Institute of Molecular Genetics, National Research Council of Italy, Pavia, Italy; 288 Human Genetics Center and Department of Epidemiology, University of Texas Health Science Center at Houston, Houston, TX USA; 289 Department of Nephrology and Rheumatology, Kliniken Südostbayern, Regensburg, Germany; 290 KfH Kidney Center Traunstein, Traunstein, Germany; 291 Center for Genomics and Personalized Medicine (CGPM), Aarhus University, Aarhus, Denmark; 292 Bioinformatics Research Centre, Aarhus University, Aarhus, Denmark; 293 USC-Office of Population Studies Foundation, University of San Carlos, Cebu City, Philippines; 294 Department of Nutrition and Dietetics, University of San Carlos, Cebu City, Philippines; 295 Human Genomics Laboratory, Pennington Biomedical Research Center, Baton Rouge, LA USA; 296 Department of Biochemistry, Wake Forest School of Medicine, Medical Center Boulevard, Winston-Salem, NC USA; 297 Department of Clinical Biochemistry, Lillebaelt Hospital, Vejle, Denmark; 298 Institute of Regional Health Research, University of Southern Denmark, Odense, Denmark; 299 Clinic of Medicine, St. Olavs Hospital, Trondheim University Hospital, Trondheim, Norway; 300 Lee Kong Chian School of Medicine, Nanyang Technological University, Singapore, Singapore; 301 Imperial College Healthcare NHS Trust, Imperial College London, London, UK; 302 Adjunct Faculty, JSS University Academy of Higher Education and Research (JSSAHER), JSS (Deemed to be) University, Mysuru, India; 303 Ophthalmology and Visual Sciences Academic Clinical Program (Eye ACP), Duke-NUS Medical School, Singapore, Singapore; 304 Department of Medical Genetics, Oslo University Hospital, Oslo, Norway; 305 Department of Medical Research, Bærum Hospital, Vestre Viken Hospital Trust, Gjettum, Norway; 306 Department of Neurology, Division of Vascular Neurology, University of Maryland School of Medicine, Baltimore, MD USA; 307 Baltimore Veterans Affairs Medical Center, Department of Neurology, Baltimore, MD USA; 308 Unidad de Investigación Médica en Bioquímica, Hospital de Especialidades, Centro Médico Nacional Siglo XXI, Instituto Mexicano del Seguro Social, Mexico City, Mexico; 309 Dipartimento di Scienze Biomediche, Università degli Studi di Sassari, Sassari, Italy; 310 Intermountain Heart Institute, Intermountain Medical Center, Murray, UT USA; 311 Department of Surgery, University of Pennsylvania, Philadelphia, PA USA; 312 Corporal Michael J. Crescenz VA Medical Center, Philadelphia, PA USA; 313 Department of Vascular Surgery, University Medical Center Utrecht, University of Utrecht, Utrecht, The Netherlands; 314 Department of Human Nutrition, Wageningen University, Wageningen, The Netherlands; 315 Center for Translational and Computational Neuroimmunology, Department of Neurology, Columbia University Medical Center, New York, NY USA; 316 Department of Oncology, University of Cambridge, Cambridge, UK; 317 Department of General Practice, Amsterdam Public Health Institute, Amsterdam UMC, location VUmc, Amsterdam, The Netherlands; 318 Department of Nutrition, Harvard T.H. Chan School of Public Health, Boston, MA USA; 319 Cardiac Arrhythmia Service, Massachusetts General Hospital, Boston, MA USA; 320 Cardiovascular Research Center, Massachusetts General Hospital, Boston, MA USA; 321 Department of Clinical Sciences in Malmö, Division of Geriatric Medicine, Lund University, Malmö, Sweden; 322 Molecular Cardiology Division, Victor Chang Cardiac Research Institute, Darlinghurst, New South Wales Australia; 323 Cardiology Department, St Vincent’s Hospital, Darlinghurst, New South Wales Australia; 324 Faculty of Medicine, UNSW Sydney, Kensington, New South Wales Australia; 325 Translational Gerontology Branch, National Institute on Aging, National Institutes of Health, Baltimore, MD USA; 326 Robertson Center for Biostatistics, University of Glasgow, Glasgow, UK; 327 Human Genetics Center, School of Public Health, University of Texas Health Science Center at Houston, Houston, TX USA; 328 Department of Public Health and Clinical Medicine, Umeå University, Umeå, Sweden; 329 Department of Internal Medicine, Wake Forest School of Medicine, Medical Center Boulevard, Winston-Salem, NC USA; 330 Faculty of Veterinary and Agricultural Science, University of Melbourne, Parkville, Victoria Australia; 331 Agriculture Victoria Research, Department of Jobs, Precincts and Regions, Bundoora, Victoria Australia; 332 Injury Prevention Research Center, University of North Carolina at Chapel Hill, Chapel Hill, NC USA; 333 Division of Physical Therapy, University of North Carolina at Chapel Hill, Chapel Hill, NC USA; 334 Centro de Investigacion en Salud Poblacional Instituto Nacional de Salud Publica and Centro de Estudios en Diabetes, Cuernavaca, Mexico; 335 Division of Human Genetics, Children’s Hospital of Philadelphia, Philadelphia, PA USA; 336 Departments of Pediatrics and Genetics, Perelman School of Medicine, University of Pennsylvania, Philadelphia, PA USA; 337 Division of Endocrinology and Diabetes, Children’s Hospital of Philadelphia, Philadelphia, PA USA; 338 Faculty of Medicine, University of Iceland, Reykjavik, Iceland; 339 Department of Preventive Medicine, Keck School of Medicine of USC, Los Angeles, CA USA; 340 Department of Pediatrics, Perelman School of Medicine, University of Pennsylvania, Philadelphia, PA USA; 341 Division of Pulmonary Medicine, Children’s Hospital of Philadelphia, Philadelphia, PA USA; 342 Institute of Biomedical and Clinical Science, University of Exeter Medical School, Exeter, UK; 343 Cardiovascular Health Research Unit, Department of Epidemiology, University of Washington, Seattle, WA USA; 344 Department of Paediatrics, Yong Loo Lin School of Medicine, National University of Singapore, Singapore, Singapore; 345 Khoo Teck Puat - National University Children’s Medical Institute, National University Health System, Singapore, Singapore; 346 Department of Internal Medicine II, Division of Cardiology, Medical University of Vienna, Vienna, Austria; 347 Menzies Research Institute Tasmania, University of Tasmania, Hobart, Tasmania Australia; 348 Centre for Eye Research Australia, Royal Victorian Eye and Ear Hospital, University of Melbourne, Melbourne, Victoria Australia; 349 Lions Eye Institute, Centre for Ophthalmology and Vision Science, University of Western Australia, Perth, Western Australia Australia; 350 Cardiology Division, Massachusetts General Hospital, Boston, MA USA; 351 Department of Genetics, Shanghai-MOST Key Laboratory of Heath and Disease Genomics, Chinese National Human Genome Center and Shanghai Industrial Technology Institute, Shanghai, China; 352 Department of Internal Medicine, University of Utah, Salt Lake City, UT USA; 353 Australian Centre for Precision Health, Clinical and Health Sciences, University of South Australia, Adelaide, South Australia Australia; 354 South Australian Health and Medical Research Institute, Adelaide, South Australia Australia; 355 Department of Environmental and Preventive Medicine, Jichi Medical University School of Medicine, Shimotsuke, Japan; 356 Department of Epidemiology and Population Health, Albert Einstein College of Medicine, Bronx, NY USA; 357 Division of Endocrinology, Diabetes and Metabolism, School of Medicine, Ohio State University, Columbus, OH USA; 358 Center for Life Course Health Research, Faculty of Medicine, University of Oulu, Oulu, Finland; 359 Unit of Primary Health Care, Oulu University Hospital, OYS, Oulu, Finland; 360 Department of Life Sciences, College of Health and Life Sciences, Brunel University London, Uxbridge, UK; 361 The Eye Hospital, School of Ophthalmology and Optometry, Wenzhou Medical University, Wenzhou, China; 362 Einthoven Laboratory for Experimental Vascular Medicine, LUMC, Leiden, The Netherlands; 363 Netherlands Heart Institute, Utrecht, The Netherlands; 364 Department of Clinical Physiology, Tampere University Hospital, Tampere, Finland; 365 Department of Clinical Physiology, Finnish Cardiovascular Research Center - Tampere, Faculty of Medicine and Health Technology, Tampere University, Tampere, Finland; 366 Laboratory of Complex Trait Genomics, Department of Computational Biology and Medical Sciences, Graduate School of Frontier Sciences, The University of Tokyo, Tokyo, Japan; 367 Department of Ophthalmology, The Catholic University of Korea Incheon St. Mary’s Hospital, Incheon, Republic of Korea; 368 NIHR Oxford Biomedical Research Centre, Churchill Hospital, Oxford, UK; 369 German Centre for Cardiovascular Research (DZHK), partner site Munich Heart Alliance, Munich, Germany; 370 Radboud University Medical Center, Radboud Institute for Health Sciences, Department of Urology, Nijmegen, The Netherlands; 371 Corneal Dystrophy Research Institute, Yonsei University College of Medicine, Seoul, Republic of Korea; 372 Saevit Eye Hospital, Goyang, Republic of Korea; 373 Department of Biochemistry, College of Medicine, Ewha Womans University, Seoul, Republic of Korea; 374 Department of Cardiology, University Heart and Vascular Center UKE Hamburg, Hamburg, Germany; 375 Institute of Cardiovascular Sciences, College of Medical and Dental Sciences, University of Birmingham, Birmingham, UK; 376 German Center for Cardiovascular Research, partner site Hamburg/Kiel/Lübeck, Hamburg, Germany; 377 Atrial Fibrillation NETwork, Münster, Germany; 378 Department of Epidemiology and Public Health, UCL Institute of Epidemiology and Health Care, University College London, London, UK; 379 Healthy Longevity Translational Research Programme, Yong Loo Lin School of Medicine, National University of Singapore, Singapore, Singapore; 380 University of Helsinki and Department of Medicine, Helsinki University Hospital, Helsinki, Finland; 381 Minerva Foundation Institute for Medical Research, Helsinki, Finland; 382 Department of Preventive Cardiology, Lipoprotein Apheresis Unit and Lipid Disorders Clinic, Metropolitan Hospital, Athens, Greece; 383 MRC-PHE Centre for Environment and Health, Imperial College London, London, UK; 384 National Heart and Lung Institute, Imperial College London, London, UK; 385 Medical Department III – Endocrinology, Nephrology, Rheumatology, University of Leipzig Medical Center, Leipzig, Germany; 386 Institute for Social and Economic Research, University of Essex, Colchester, UK; 387 Institute of Clinical Medicine, Internal Medicine, University of Eastern Finland and Kuopio University Hospital, Kuopio, Finland; 388 Department of Medicine, University of Colorado at Denver, Aurora, CO USA; 389 Berlin Institute of Health at Charité – Universitätsmedizin Berlin, Berlin, Germany; 390 Epidemiology Program, University of Hawaii Cancer Center, Honolulu, HI USA; 391 Department of Internal Medicine, Ewha Womans University School of Medicine, Seoul, Republic of Korea; 392 Department of Epidemiology and Biostatistics, Peking University Health Science Center, Beijing, China; 393 Peking University Center for Public Health and Epidemic Preparedness and Response, Beijing, China; 394 Institute of Epidemiology and Biobank Popgen, Kiel University, Kiel, Germany; 395 Key Laboratory of Systems Health Science of Zhejiang Province, Hangzhou Institute for Advanced Study, University of Chinese Academy of Sciences, Chinese Academy of Sciences, Hangzhou, China; 396 Department of Clinical Medicine, Faculty of Health and Medical Sciences, University of Copenhagen, Copenhagen, Denmark; 397 Division of Cardiovascular Medicine and Abboud Cardiovascular Research Center, University of Iowa Hospitals and Clinics, Iowa City, IA USA; 398 Alliance for Human Development, Lunenfeld-Tanenbaum Research Institute, Sinai Health System, Toronto, Ontario Canada; 399 Department of Medical Epidemiology and Biostatistics, Karolinska Institutet, Stockholm, Sweden; 400 Division of Cardiology, University of California, San Francisco, San Francisco, CA USA; 401 Department of Medicine, Internal Medicine, Lausanne University Hospital, Lausanne, Switzerland; 402 University of Lausanne, Lausanne, Switzerland; 403 SYNLAB Academy, SYNLAB Holding Deutschland, Mannheim, Germany; 404 Clinical Institute of Medical and Chemical Laboratory Diagnostics, Medical University of Graz, Graz, Austria; 405 Department of Medicine, Division of Cardiology, Duke University School of Medicine, Durham, NC USA; 406 Duke Molecular Physiology Institute, Duke University School of Medicine, Durham, NC USA; 407 Psychiatric Genetics, QIMR Berghofer Medical Research Institute, Brisbane, Queensland Australia; 408 Geriatric Medicine, Institute of Medicine, Sahlgrenska Academy, University of Gothenburg, Gothenburg, Sweden; 409 Geriatrics Research and Education Clinical Center, Baltimore Veterans Administration Medical Center, Baltimore, MD USA; 410 Centre for Vision Research and Department of Ophthalmology, Westmead Millennium Institute of Medical Research, University of Sydney, Sydney, New South Wales Australia; 411 Department of Public Health and Primary Care, Leiden University Medical Center, Leiden, The Netherlands; 412 Usher Institute of Population Health Sciences and Informatics, University of Edinburgh, Edinburgh, UK; 413 Electrophysiology Section, Division of Cardiovascular Medicine, Perelman School of Medicine, University of Pennsylvania, Philadelphia, PA USA; 414 Department of Medicine, University of North Carolina at Chapel Hill, Chapel Hill, NC USA; 415 Department of Chronic Diseases, Norwegian Institute of Public Health, Oslo, Norway; 416 Department of Pain Management and Research, Oslo University Hospital, Oslo, Norway; 417 Institute of Human Genetics, School of Medicine and University Hospital Bonn, Bonn, Germany; 418 Sahlgrenska University Hospital, Department of Drug Treatment, Gothenburg, Sweden; 419 Laboratorio de Inmunogenómica y Enfermedades Metabólicas, Instituto Nacional de Medicina Genómica, CDMX, Mexico City, Mexico; 420 Paavo Nurmi Centre, Sports and Exercise Medicine Unit, Department of Physical Activity and Health, University of Turku, Turku, Finland; 421 Institute for Precision Health, David Geffen School of Medicine at UCLA, Los Angeles, CA USA; 422 Pat MacPherson Centre for Pharmacogenetics and Pharmacogenomics, Division of Population Health and Genomics, School of Medicine, University of Dundee, Ninewells Hospital and Medical School, Dundee, UK; 423 Centre Nutrition, Santé et Société (NUTRISS), Institute of Nutrition and Functional Foods, Université Laval, Québec City, Quebec Canada; 424 IBE-Chair of Epidemiology, LMU Munich, Neuherberg, Germany; 425 Population, Policy and Practice, UCL Great Ormond Street Hospital Institute of Child Health, London, UK; 426 Department of Medicine, University of Pennsylvania, Philadelphia, PA USA; 427 Department of Clinical Physiology and Nuclear Medicine, Turku University Hospital, Turku, Finland; 428 Hero DMC Heart Institute, Dyanand Medical College, Ludhiana, India; 429 Second Department of Cardiology, Medical School, National and Kapodistrian University of Athens, University General Hospital Attikon, Athens, Greece; 430 Division of Biostatistics, Washington University School of Medicine, St Louis, MO USA; 431 Genetics, Merck Sharp & Dohme, Kenilworth, NJ USA; 432 Department of Epidemiology, University of Washington, Seattle, WA USA; 433 Department of Endocrinology and Metabolism, Kyung Hee University School of Medicine, Seoul, Korea; 434 Department of Public Health, Clinicum, Faculty of Medicine, University of Helsinki, Helsinki, Finland; 435 Departments of Medicine, Pharmacology, and Biomedical Informatics, Vanderbilt University Medical Center, Nashville, TN USA; 436 Department of Epidemiology and Data Science, Amsterdam Public Health Institute, Amsterdam Cardiovascular Sciences Institute, Amsterdam UMC, location VUmc, Amsterdam, The Netherlands; 437 Department of Cardiology and Department of Medicine, Columbia University, New York, NY USA; 438 Department of Pediatrics, Section of Genetics, College of Medicine, University of Oklahoma Health Sciences Center, Oklahoma City, OK USA; 439 Department of Pharmaceutical Sciences, University of Oklahoma Health Sciences Center, Oklahoma City, OK USA; 440 Department of Physiology, University of Oklahoma Health Sciences Center, Oklahoma City, OK USA; 441 Oklahoma Center for Neuroscience, University of Oklahoma Health Sciences Center, Oklahoma City, OK USA; 442 Institute of Cardiovascular and Medical Sciences, University of Glasgow, Glasgow, UK; 443 Gottfried Schatz Research Center (for Cell Signaling, Metabolism and Aging), Medical University of Graz, Graz, Austria; 444 Institute of Nutritional Science, University of Potsdam, Nuthetal, Germany; 445 Deutsches Herzzentrum München, Cardiology, Deutsches Zentrum für Herz- und Kreislaufforschung (DZHK) – Munich Heart Alliance, and Technische Universität München, München, Germany; 446 School of Biomedical Science and Pharmacy, University of Newcastle, New Lambton Heights, New South Wales Australia; 447 Department of Medicine, Vanderbilt University Medical Center, Nashville, TN USA; 448 Central University of Punjab, Bathinda, India; 449 Department of Medicine I, University Hospital, LMU Munich, Munich, Germany; 450 Department of Cardiology, Clinical Sciences, Lund University and Skåne University Hospital, Lund, Sweden; 451 The Wallenberg Laboratory, Department of Molecular and Clinical Medicine, Institute of Medicine, Gothenburg University and the Department of Cardiology, Sahlgrenska University Hospital, Gothenburg, Sweden; 452 Wallenberg Center for Molecular Medicine and Lund University Diabetes Center, Lund University, Lund, Sweden; 453 Population Health Research Institute, St George’s, University of London, London, UK; 454 Molecular Epidemiology Section, Department of Biomedical Data Sciences, Leiden University Medical Center, Leiden, The Netherlands; 455 Department of Genetics, Stanford University School of Medicine, Stanford, CA USA; 456 Department of Medicine, Faculty of Medicine, Université de Montréal, Montreal, Quebec Canada; 457 Helsinki University Central Hospital, Research Program for Clinical and Molecular Metabolism, University of Helsinki, Helsinki, Finland; 458 Folkhälsan Research Center, Helsinki, Finland; 459 Department of Public Health, University of Helsinki, Helsinki, Finland; 460 Diabetes Research Group, King Abdulaziz University, Jeddah, Saudi Arabia; 461 Unidad de Biología Molecular y Medicina Genómica, Instituto de Investigaciones Biomédicas, UNAM, Mexico City, Mexico; 462 Instituto Nacional de Ciencias Médicas y Nutrición Salvador Zubirán, Mexico City, Mexico; 463 Yong Loo Lin School of Medicine, National University of Singapore and National University Health System, Singapore, Singapore; 464 Milken Institute School of Public Health, The George Washington University, Washington, DC USA; 465 Department Geriatric Medicine, Amsterdam Public Health, Amsterdam UMC location University of Amsterdam, Amsterdam, The Netherlands; 466 Department of Epidemiology and Data Science, Amsterdam UMC, Amsterdam, The Netherlands; 467 Interfaculty Institute for Genetics and Functional Genomics, University Medicine Greifswald, Greifswald, Germany; 468 Unidad de Investigación Médica en Epidemiología Clínica, Hospital de Especialidades, Centro Médico Nacional Siglo XXI, Instituto Mexicano del Seguro Social, Mexico City, Mexico; 469 Institute of Cellular Medicine, Newcastle University, Newcastle upon Tyne, UK; 470 Department of Population and Public Health Sciences, Keck School of Medicine of USC, Los Angeles, CA USA; 471 Department of Physiology and Neuroscience, Keck School of Medicine of USC, Los Angeles, CA USA; 472 USC Diabetes and Obesity Research Institute, Keck School of Medicine of USC, Los Angeles, CA USA; 473 Lundbeck Foundation Center for GeoGenetics, GLOBE Institute, University of Copenhagen, Copenhagen, Denmark; 474 School of Chinese Medicine, China Medical University, Taichung, Taiwan; 475 Diabetes Unit, KEM Hospital and Research Centre, Pune, India; 476 Kurume University School of Medicine, Kurume, Japan; 477 Division of Cancer Control and Population Sciences, UPMC Hillman Cancer Center, University of Pittsburgh, Pittsburgh, PA USA; 478 Department of Epidemiology, Graduate School of Public Health, University of Pittsburgh, Pittsburgh, PA USA; 479 TUM School of Medicine, Technical University of Munich and Klinikum Rechts der Isar, Munich, Germany; 480 Institute of Clinical Medicine, Faculty of Medicine, University of Oslo, Oslo, Norway; 481 Department of Population Health Sciences, Geisinger, Danville, PA USA; 482 Vanderbilt Genetics Institute, Division of Genetic Medicine, Vanderbilt University Medical Center, Nashville, TN USA; 483 Department of Medicine, Veterans Affairs Boston Healthcare System, Boston, MA USA; 484 Department of Epidemiology, Emory University Rollins School of Public Health, Atlanta, GA USA; 485 Atlanta VA Health Care System, Decatur, GA USA; 486 Princess Al-Jawhara Al-Brahim Centre of Excellence in Research of Hereditary Disorders (PACER-HD), King Abdulaziz University, Jeddah, Saudi Arabia; 487 The Mindich Child Health and Development Institute, Icahn School of Medicine at Mount Sinai, New York, NY USA; 488 School of Life Sciences, Westlake University, Hangzhou, China; 489 Westlake Laboratory of Life Sciences and Biomedicine, Hangzhou, China; 490 Department of Human Genetics, University of Michigan, Ann Arbor, MI USA; 491 McDonnell Genome Institute and Department of Medicine, Washington University School of Medicine, St Louis, MO USA; 492 Laboratory of Statistical Immunology, Immunology Frontier Research Center (WPI-IFReC), Osaka, Japan; 493 Integrated Frontier Research for Medical Science Division, Institute for Open and Transdisciplinary Research Initiatives, Osaka University, Osaka, Japan; 494 Programs in Metabolism and Medical and Population Genetics, Broad Institute of MIT and Harvard, Cambridge, MA USA; 495 Departments of Pediatrics and Genetics, Harvard Medical School, Boston, MA USA; 496 Present Address: Department of Mathematics and Statistics, St Cloud State University, St Cloud, MN USA; 497 Genentech, South San Francisco, CA USA; 498 Present Address: Laboratory for Systems Genetics, RIKEN Center for Integrative Medical Sciences, Kanagawa, Japan; 499 Department of Genome Informatics, Graduate School of Medicine, The University of Tokyo, Tokyo, Japan

**Keywords:** Genome-wide association studies, Quantitative trait, Genetic markers

## Abstract

Common single-nucleotide polymorphisms (SNPs) are predicted to collectively explain 40–50% of phenotypic variation in human height, but identifying the specific variants and associated regions requires huge sample sizes^1^. Here, using data from a genome-wide association study of 5.4 million individuals of diverse ancestries, we show that 12,111 independent SNPs that are significantly associated with height account for nearly all of the common SNP-based heritability. These SNPs are clustered within 7,209 non-overlapping genomic segments with a mean size of around 90 kb, covering about 21% of the genome. The density of independent associations varies across the genome and the regions of increased density are enriched for biologically relevant genes. In out-of-sample estimation and prediction, the 12,111 SNPs (or all SNPs in the HapMap 3 panel^2^) account for 40% (45%) of phenotypic variance in populations of European ancestry but only around 10–20% (14–24%) in populations of other ancestries. Effect sizes, associated regions and gene prioritization are similar across ancestries, indicating that reduced prediction accuracy is likely to be explained by linkage disequilibrium and differences in allele frequency within associated regions. Finally, we show that the relevant biological pathways are detectable with smaller sample sizes than are needed to implicate causal genes and variants. Overall, this study provides a comprehensive map of specific genomic regions that contain the vast majority of common height-associated variants. Although this map is saturated for populations of European ancestry, further research is needed to achieve equivalent saturation in other ancestries.

## Abstract (web summary)

A large genome-wide association study of more than 5 million individuals reveals that 12,111 single-nucleotide polymorphisms account for nearly all the heritability of height attributable to common genetic variants.

## Main

Since 2007, genome-wide association studies (GWASs) have identified thousands of associations between common SNPs and height, mainly using studies with participants of European ancestry. The largest GWAS published so far for adult height focused on common variation and reported up to 3,290 independent associations in 712 loci using a sample size of up to 700,000 individuals^3^. Adult height, which is highly heritable and easily measured, has provided a larger number of common genetic associations than any other human phenotype. In addition, a large collection of genes has been implicated in disorders of skeletal growth, and these are enriched in loci mapped by GWASs of height in the normal range. These features make height an attractive model trait for assessing the role of common genetic variation in defining the genetic and biological architecture of polygenic human phenotypes.

As available sample sizes continue to increase for GWASs of common variants, it becomes important to consider whether these larger samples can ‘saturate’ or nearly completely catalogue the information that can be derived from GWASs. This question of completeness can take several forms, including prediction accuracy compared with heritability attributable to common variation, the mapping of associated genomic regions that account for this heritability, and whether increasing sample sizes continue to provide additional information about the identity of prioritized genes and gene sets. Furthermore, because most GWASs continue to be performed largely in populations of European ancestry, it is necessary to address these questions of completeness in the context of multiple ancestries. Finally, some have proposed that, when sample sizes become sufficiently large, effectively every gene and genomic region will be implicated by GWASs, rather than certain subsets of genes and biological pathways being specified^4^.

Here, using data from 5.4 million individuals, we set out to map common genetic associations with adult height, using variants catalogued in the HapMap 3 project (HM3), and to assess the saturation of this map with respect to variants, genomic regions and likely causal genes and gene sets. We identify significant variants, examine signal density across the genome, perform out-of-sample estimation and prediction analyses within studies of individuals of European ancestry and other ancestries and prioritize genes and gene sets as likely mediators of the effects on height. We show that this set of common variants reaches predicted limits for prediction accuracy within populations of European ancestry and largely saturates both the genomic regions associated with height and broad categories of gene sets that are likely to be relevant; future work will be required to extend prediction accuracy to populations of other ancestries, to account for rarer genetic variation and to more definitively connect associated regions with individual probable causal genes and variants.

**Extended Data Fig. 1:**
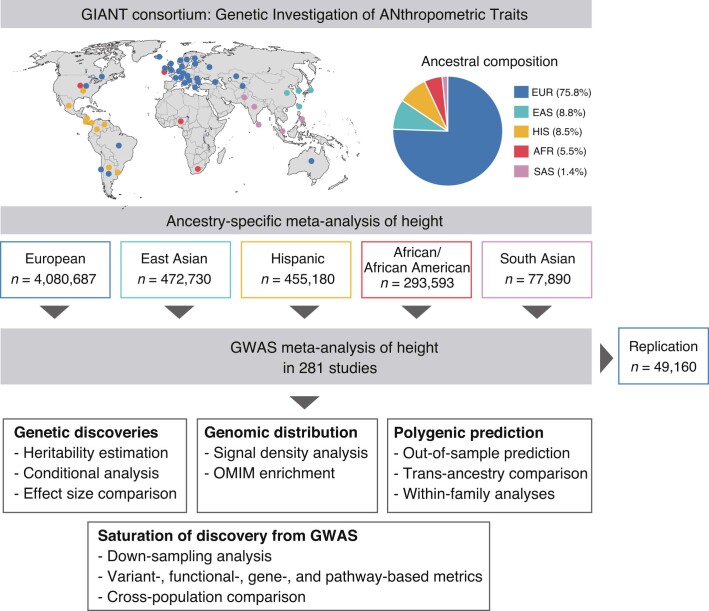
Broad ancestries composition. Geographical mapping and ancestries composition of 281 studies meta-analysed in this study. Various analyses were performed including (1) dectection of height-associated SNPs (Genetic discoveries box), (2) quantification of the genomic distribution of height-associated loci (Genomic distribution box), (3) assessement of the performances of polygenic predictors of height (Polygenic prediction box), and (4) assessment of the relationship between GWAS sample size and discoveries (Saturation of discovery from GWAS box).

An overview of our study design and analysis strategy is provided in Extended Data Fig. 1.

## Meta-analysis identifies 12,111 height-associated SNPs

We performed genetic analysis of up to 5,380,080 individuals from 281 studies from the GIANT consortium and 23andMe. Supplementary Fig. 1 represents projections of these 281 studies onto principal components reflecting differences in allele frequencies across ancestry groups in the 1000 Genomes Project (1KGP)^5^. Altogether, our discovery sample includes 4,080,687 participants of predominantly European ancestries (75.8% of total sample); 472,730 participants with predominantly East Asian ancestries (8.8%); 455,180 participants of Hispanic ethnicity with typically admixed ancestries (8.5%); 293,593 participants of predominantly African ancestries—mostly African American individuals with admixed African and European ancestries (5.5%); and 77,890 participants of predominantly South Asian ancestries (1.4%). We refer to these five groups of participants or cohorts as EUR, EAS, HIS, AFR and SAS, respectively, while recognizing that these commonly used groupings oversimplify the actual genetic diversity among participants. Cohort-specific information is provided in Supplementary Tables 1–3. We tested the association between standing height and 1,385,132 autosomal bi-allelic SNPs from the HM3 tagging panel^2^, which contains more than 1,095,888 SNPs with a minor allele frequency (MAF) greater than 1% in each of the five ancestral groups included in our meta-analysis. Supplementary Fig. 2 shows the frequency and imputation quality distribution of HM3 SNPs across all five groups of cohorts.

We first performed separate meta-analyses in each of the five groups of cohorts. We identified 9,863, 1,511, 918, 453 and 69 quasi-independent genome-wide significant (GWS; *P* < 5 × 10^−8^) SNPs in the EUR, HIS, EAS, AFR and SAS groups, respectively (Table 1 and Supplementary Tables 4–8). Quasi-independent associations were obtained after performing approximate conditional and joint (COJO) multiple-SNP analyses^6^, as implemented in GCTA^7^ (Methods). Supplementary Note 1 presents sensitivity analyses of these COJO results, highlights biases due to relatively long-range linkage disequilibrium (LD) in admixed AFR and HIS individuals^8^ (Supplementary Fig. 3), and shows how to correct those biases by varying the GCTA input parameters (Supplementary Fig. 4). Moreover, previous studies have shown that confounding due to population stratification may remain uncorrected in large GWAS meta-analyses^9,10^. Therefore, we specifically investigated confounding effects in all ancestry-specific GWASs, and found that our results are minimally affected by population stratification (Supplementary Note 2 and Supplementary Figs. 5–7).

**Table 1:** Table 1 Summary of results from within-ancestry and trans-ancestry GWAS meta-analyses

Cohort ancestry or ethnic group	Number of studies	Max *n* (mean *n*)	Number of GWS COJO SNPs (*P*_GWAS_ < 5 × 10^−8^)	Number of GWS loci (35 kb)	Cumulative length of non-overlapping GWS loci in Mb (% of genome)
European (EUR)	173	4,080,687 (3,612,229)	9,863 (8,382)	6,386	552.5 (18.4%)
East Asian (EAS)	56	472,730 (320,570)	918 (807)	821	60.5 (2.0%)
Hispanic (HIS)	11	455,180 (431,645)	1,511 (1,195)	1,373	101.0 (3.3%)
African (AFR)	29	293,593 (222,981)	453 (404)	412	30.4 (1.0%)
South Asian (SAS)	12	77,890 (59,420)	69 (65)	66	4.7 (0.2%)
Trans-ancestry meta-analysis (META_FE_)	281	5,314,291* (4,611,160)	12,111 (9,920)	7,209	647.5 (21.6%)

*n* denotes the sample size for each SNP. GWS: genome-wide significant (*P* < 5 × 10^−8^). COJO SNPs: near-independent GWS SNPs identified using an approximate COJO analysis implemented in the GCTA software. *P*_GWAS_: *P* value from a marginal association test. GWS loci were defined as genomic regions centred around each GWS SNP and including all SNPs within 35 kb on each side of the lead GWS SNP. Overlapping GWS loci were merged so that the number and cumulative length of GWS loci are calculated on non-overlapping GWS loci. The percentage of the genome covered was calculated by dividing the cumulative of GWS loci by 3,039 Mb (the approximated length of the human genome).

*The number of individuals in the trans-ancestry meta-analysis (*n* = 5,314,291) is smaller than the sum of ancestry-group-specific meta-analyses (*n* = 5,380,080) because of variation in per-SNP sample sizes for SNPs included in the final analysis.

**Extended Data Fig. 2:**
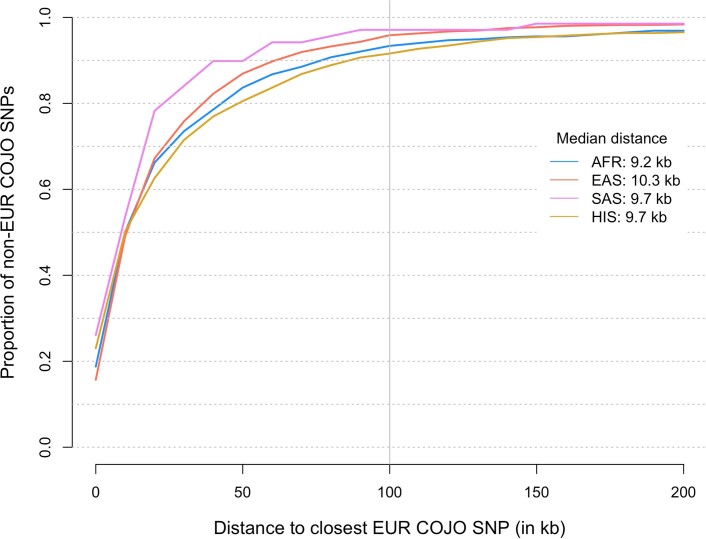
Colocalization of height-associated signals across ancestries. Proportion (y-axis) of GWS SNPs identified in our GWAS meta-analyses of non-European (non-EUR: African – AFR; East Asian – EAS; South Asian – SAS; Hispanic – HIS) ancestry/ethnicity participants thar are located within a certain distance (x-axis) of GWS SNPs identified in our GWAS meta-analysis of EUR participants only. Source data

To compare results across the five groups of cohorts, we examined the genetic and physical colocalization between SNPs identified in the largest group (EUR) with those found in the other (non-EUR) groups. We found that more than 85% of GWS SNPs detected in the non-EUR groups are in strong LD (${r}_{{\rm{LD}}}^{2}$ > 0.8) with at least one variant reaching marginal genome-wide significance (*P*_GWAS_ < 5 × 10^−8^) in EUR (Supplementary Tables 5–8). Furthermore, more than 91% of associations detected in non-EUR meta-analyses fall within 100 kb of a GWS SNP identified in EUR (Extended Data Fig. 2). By contrast, a randomly sampled HM3 SNP (matched with GWS SNPs identified in non-EUR meta-analyses on 24 functional annotations; Methods) falls within 100 kb of a EUR GWS SNP 55% of the time on average (s.d. = 1% over 1,000 draws). Next, we quantified the cross-ancestry correlation of marginal allele substitution effects (*ρ*_b_) at GWS SNPs for all pairs of ancestry groups. We estimated *ρ*_b_ using five subsets of GWS SNPs identified in each of the ancestry groups, which also reached marginal genome-wide significance in at least one group. After correction for winner’s curse^11,12^, we found that *ρ*_b_ ranged between 0.64 and 0.99 across all pairs of ancestry groups and all sets of GWS SNPs (Supplementary Figs. 8–12). We also extended the estimation of *ρ*_b_ for SNPs that did not reach genome-wide significance and found that *ρ*_b_ > 0.5 across all comparisons (Supplementary Fig. 13). Thus, the observed GWS height associations are substantially shared across major ancestral groups, consistent with previous studies based on smaller sample sizes^13,14^.

To find signals that are specific to certain groups, we tested whether any individual SNPs detected in non-EUR GWASs are conditionally independent of signals detected in EUR GWASs. We fitted an approximate joint model that includes GWS SNPs identified in EUR and non-EUR, using LD reference panels specific to each ancestry group. After excluding SNPs in strong LD (${r}_{{\rm{LD}}}^{2}$ > 0.8 in either ancestry group), we found that 2, 17, 49 and 63 of the GWS SNPs detected in SAS, AFR, EAS and HIS GWASs, respectively, are conditionally independent of GWS SNPs identified in EUR GWASs (Supplementary Table 9). On average, these conditionally independent SNPs have a larger MAF and effect size in non-EUR than in EUR cohorts, which may have contributed to an increased statistical power of detection. The largest frequency difference relative to EUR was observed for rs2463169 (height-increasing G allele frequency: 23% in AFR versus 84% in EUR) within the intron of *PAWR*, which encodes the prostate apoptosis response-4 protein. Of note, rs2463169 is located within the 12q21.2 locus, where a strong signal of positive selection in West African Yoruba populations was previously reported^15^. The estimated effect at rs2463169 is *β* ≈ 0.034 s.d. per G allele in AFR versus *β* ≈ −0.002 s.d. per G allele in EUR, and the *P* value of marginal association in EUR is *P*_EUR_ = 0.08, suggesting either a true difference in effect size or nearby causal variant(s) with differing LD to rs2463169.

Given that our results show a strong genetic overlap of GWAS signals across ancestries, we performed a fixed-effect meta-analysis of all five ancestry groups to maximize statistical power for discovering associations due to shared causal variants. The mean Cochran’s heterogeneity *Q*-statistic is around 34% across SNPs, which indicates moderate heterogeneity of SNP effects between ancestries. The mean chi-square association statistic in our fixed-effect meta-analysis (hereafter referred to as META_FE_) is around 36, and around 18% of all HM3 SNPs are marginally GWS. Moreover, we found that allele frequencies in our META_FE_ were very similar to that of EUR (mean fixation index of genetic differentiation (*F*_ST_) across SNPs between EUR and META_FE_ is around 0.001), as expected because our META_FE_ consists of more than 75% EUR participants and around 14% participants with admixed European and non-European ancestries that is, HIS and AFR). To further assess whether LD in our META_FE_ could be reasonably approximated by the LD from EUR, we performed an LD score regression^16^ analysis of our META_FE_ using LD scores estimated in EUR. In this analysis, we focused on the attenuation ratio statistic (*R*_LDSC-EUR_), for which large values can also indicate strong LD inconsistencies between a given reference and GWAS summary statistics. A threshold of *R*_LDSC_ > 20% was recommended by the authors of the LDSC software as a rule-of-thumb to detect such inconsistencies. Using EUR LD scores in the GWAS of HIS, which is the non-EUR group that is genetically closest to EUR (*F*_ST_ ≈ 0.02), yields an estimated *R*_LDSC-EUR_ of around 25% (standard error (s.e.) 1.8%), consistent with strong LD differences between HIS and EUR. By contrast, in our META_FE_, we found an estimated *R*_LDSC-EUR_ of around 4.5% (s.e. 0.8%), which is significantly lower than 20% and not statistically different from 3.8% (s.e. 0.8%) in our EUR meta-analysis. Furthermore, we show in Supplementary Note 1 that using a composite LD reference containing samples from various ancestries (with proportions matching that in our META_FE_) does not improve signal detection over using an EUR LD reference. Altogether, these analyses suggest that LD in our META_FE_ can be reasonably approximated by LD from EUR.

**Fig. 1:**
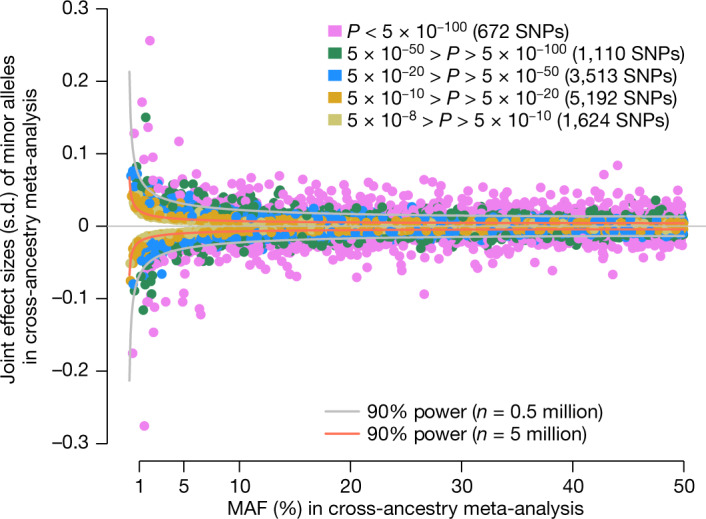
Relationship between frequency and estimated effect sizes of minor alleles. Each dot represents one of the 12,111 quasi-independent GWS SNPs that were identified in our cross-ancestry GWAS meta-analysis. Data underlying this figure are available in Supplementary Table 10. SNP effect estimates (*y* axis) are expressed in height standard deviation (s.d.) per minor allele as defined in our cross-ancestry GWAS meta-analysis. SNPs were stratified in five classes according to their *P* value (*P*) of association. We show two curves representing the theoretical relationship between frequency and expected magnitude of SNP effect detectable at P < 5 × 10^−8^ with a statistical power of 90%. Statistical power was assessed under two experimental designs with sample sizes equal to *n* = 0.5 million and *n* = 5 million. Source data

**Fig. 2:**
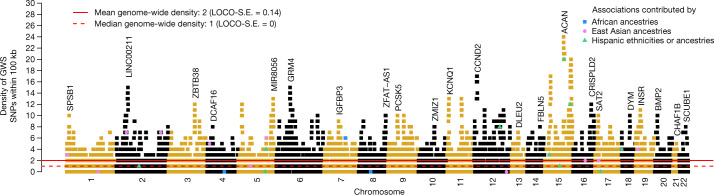
Brisbane plot showing the genomic density of independent genetic associations with height. Each dot represents one of the 12,111 quasi-independent GWS (*P* < 5 × 10^−8^) height-associated SNPs identified using approximate COJO analyses of our cross-ancestry GWAS meta-analysis. Data underlying this figure are available in Supplementary Table 10. GWS SNPs with the largest density on each chromosome were annotated with the closest gene. We highlight 24 of 12,111 associations that are mainly contributed by groups of non-European ancestry (3 from African ancestries, 10 from Hispanic ethnicities or ancestries and 11 from East Asian ancestries). The full list of height-associated SNPs detected in groups of non-European ancestry and independent of associations detected in European ancestry GWASs is reported in Supplementary Table 9. Signal density was calculated for each associated SNP as the number of other independent associations within 100 kb. A density of 1 means that a GWS COJO SNP shares its location with another independent GWS COJO SNP within less than 100 kb. The mean signal density across the genome is 2 and the median signal density is 1 (s.e. 0.14 and 0.0, respectively). The s.e. values were calculated using a leave-one-chromosome-out jackknife approach (LOCO-S.E.). SNPs that did not reach genome-wide significance  are not represented on the figure. Source data

We therefore proceeded to identify quasi-independent GWS SNPs from the multi-ancestry meta-analysis by performing a COJO analysis of our META_FE_, using genotypes from around 350,000 unrelated EUR participants in the UK Biobank (UKB) as an LD reference. We identified 12,111 quasi-independent GWS SNPs, including 9,920 (82%) primary signals with a GWS marginal effect and 2,191 secondary signals that only reached GWS in a joint regression model (Supplementary Table 10). Figure 1 represents the relationship between frequency and joint effect sizes of minor alleles at these 12,111 associations. Of the GWS SNPs obtained from the non-EUR meta-analyses above that were conditionally independent of the EUR GWS SNPs, 0/2 in SAS, 5/17 in AFR, 27/49 in EAS and 27/63 in HIS were marginally significant in our META_FE_ (Supplementary Table 9), and 24 of those (highlighted in Fig. 2) overlapped with our list of 12,111 quasi-independent GWS SNPs.

**Extended Data Fig. 3:**
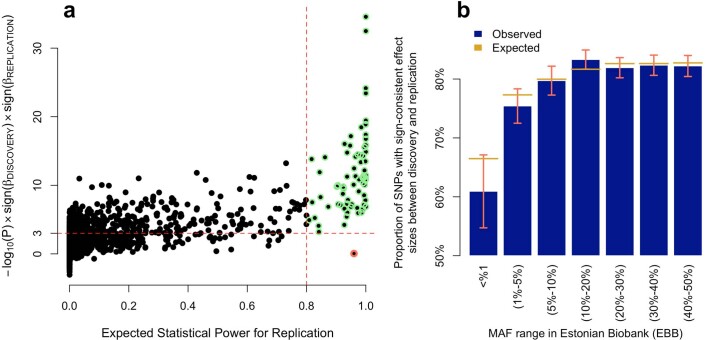
Replication of marginal associations in the EBB. **a**, Each dot represents one the 12,111 SNPs detected in our trans-ancestry meta-analysis. The x-axis represents the expected statistical power to replicate each association (*P*<0.05/9,473 = 5.3×10^−6^; where 9,473 is the number of associations reaching marginal genome-wide significance in our discovery trans-ancestry GWAS and with a minor allele frequency>1% in the EBB sample). The y-axis represents the -log_10_ of the association p-value in the EBB multiplied by the product of signs of estimated SNP effects in the discovery and in the EBB. Horizontal dotted line represents replication at *P*<0.001 and the vertical dotted line indicates 80% of statistical power. SNPs highlighted in green have an expected statistical power for replication >80%. One outlier (rs11100870), highlighted in red, does not replicate in the EBB sample. **b**, Proportion (*P*) of SNPs with a sign-consistent estimated effect between discovery GWAS (N~5.3M) and EBB. Expected proportions (*E*[*P*]) are calculated using equation (2) in the Methods. Error bars are defined as 1.96×$\sqrt{P(1-P)/m}$, where *m* is the number of SNPs in the corresponding MAF interval. Source data

We next sought to replicate the 12,111 META_FE_ signals using GWAS data from 49,160 participants in the Estonian Biobank (EBB). We first re-assessed the consistency of allele frequencies between our META_FE_ and the EBB set. We found a correlation of allele frequencies of around 0.98 between the two datasets and a mean *F*_ST_ across SNPs of around 0.005, similar to estimates that were obtained between populations from the same continent. Of the 12,111 GWS SNPs identified through our COJO analysis, 11,847 were available in the EBB dataset, 97% of which (11,529) have a MAF greater than 1% (Supplementary Table 10). Given the large difference in sample size between our discovery and replication samples, direct statistical replication of individual associations at GWS is not achievable for most SNPs identified (Extended Data Fig. 3a). Instead, we assessed the correlation of SNP effects between our discovery and replication GWASs as an overall metric of replicability^3,17^. Among the 11,529 out of 11,847 SNPs that had a MAF greater than 1% in the EBB, we found a correlation of marginal SNP effects of *ρ*_b_ = 0.93 (jackknife standard error; s.e. 0.01) and a correlation of conditional SNP effects using the same LD reference panel of *ρ*_b_ = 0.80 (s.e. 0.03; Supplementary Fig. 14). Although we had limited power to replicate associations with 238 GWS variants that are rare in the EBB (MAF < 1%), we found, consistent with expectations (Methods and Extended Data Fig. 3b), that 60% of them had a marginal SNP effect that was sign-consistent with that from our discovery GWAS (Fisher's exact test; *P* = 0.001). The proportion of sign-consistent SNP effects was greater than 75% (Fisher's exact test; *P* < 10^−50^) for variants with a MAF greater than 1%—also consistent with expectations (Extended Data Fig. 3b). Altogether, our analyses demonstrate the robustness of our findings and show their replicability in an independent sample.

## Genomic distribution of height-associated SNPs

**Extended Data Fig. 4:**
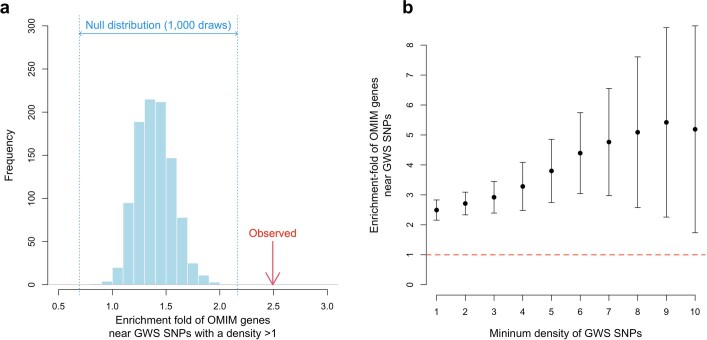
Enrichment of genes containing pathogenic mutations that cause extreme height or abnormal skeletal growth syndromes near hotspots of GWS SNPs. Four hundred and sixty-two (462) autosomal genes were curated from the Online Mendelian Inheritance in Man (OMIM) database. **a**, Red arrow indicates the observed enrichment statistic (OR = 2.5-fold) measuring the odds ratio of the presence of an OMIM gene within 100 kb of a GWS SNPs with a density > 1. The blue histogram represents the distribution of enrichment statistics from 1,000 random genes matched, which length distribution matches that of the OMIM genes. **b**, Enrichment of OMIM genes near high density GWS SNPs. High density is defined by on the x-axis by the minimum number of other independent GWS SNPs detected within 100 kb. Source data

To examine signal density among the 12,111 GWS SNPs detected in our META_FE_, we defined a measure of local density of association signals for each GWS SNP on the basis of the number of additional independent associations within 100 kb (Supplementary Fig. 15). Supplementary Fig. 16 shows the distributions of signal density for GWS SNPs identified in each ancestry group and in our META_FE_. We observed that 69% of GWS SNPs shared their location with another associated, conditionally independent, GWS SNP (Fig. 2). The mean signal density across the entire genome is 2.0 (s.e. 0.14), consistent with a non-random genomic distribution of GWS SNPs. Next, we evaluated signal density around 462 autosomal genes curated from the Online Mendelian Inheritance in Man (OMIM) database^18^ as containing pathogenic mutations that cause syndromes of abnormal skeletal growth ('OMIM genes'; Methods and Supplementary Table 11). We found that a high density of height-associated SNPs is significantly correlated with the presence of an OMIM gene nearby^19,20^ (enrichment fold of OMIM gene when density is greater than 1: 2.5×; *P* < 0.001; Methods and Extended Data Fig. 4a). Notably, the enrichment of OMIM genes almost linearly increases with the density of height-associated SNPs (Extended Data Fig. 4b). Thus, these 12,111 GWS SNPs nonrandomly cluster near each other and near known skeletal growth genes.

**Extended Data Fig. 5:**
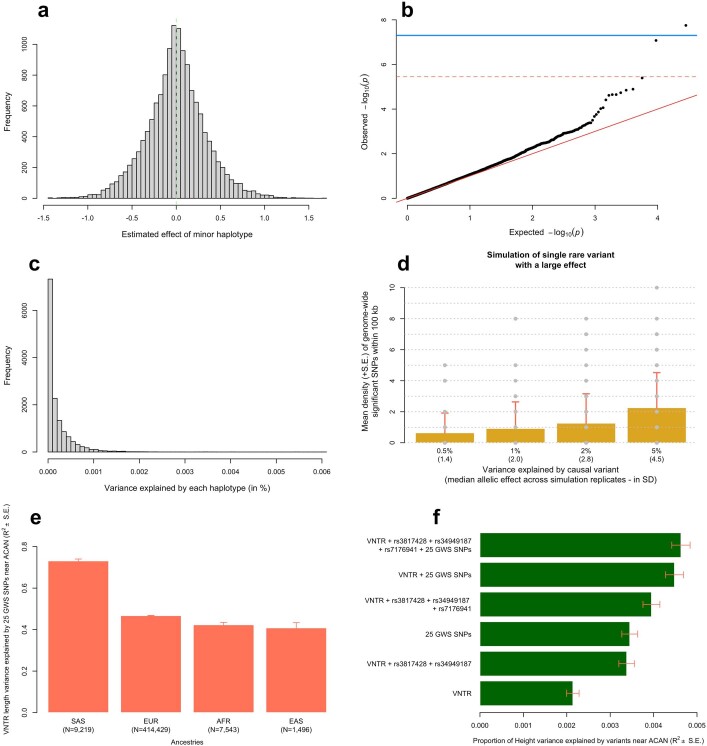
Haplotypic analysis at the *ACAN* locus. **a**, Distribution of estimated haplotype effects from 14,117 haplotypes covering a 100 kb long genomic region near the *ACAN* gene (hg19 genomic coordinates: chr15:89,307,521-89,407,521). **b**, Quantile-quantile plot of associations between these 14,117 haplotypes and height. **c**, Distribution of the variance explained by each of the 14,117 haplotypes. **d**, Mean signals density (y-axis) across simulated data where 1 causal SNP within the locus explains between 0.5% and 5% (x-axis) of trait variance. Causal variants were sampled from a pool of 13 SNPs with a 1.4×10^−5^ < MAF < 1% genotyped in 291,683 unrelated EUR participants of the UKB, with no missing values at these 13 SNPs. Standard errors were calculated as the standard deviation (s.d.) of signal density across 100 simulation replicates. GCTA-COJO analyses to identify independent signals were performed using a subset of 10,000 unrelated EUR participants of the UKB to mimic the large discrepancy between the size of the discovery GWAS and that of the LD reference used in our real data analyses. **e**, Proportion of VNTR length explained by 25 GWS SNPs identified near *ACAN* in 4 ancestries (European: EUR; South Asian: SAS; East Asian: EAS; African: AFR). **f**, Proportion of height variance explained in a sample of EUR UK Biobank participants by various sets of polymorphisms at the *ACAN* locus. rs3817428 and rs34949187 are two missense variants and rs7176941 is an intronic variant with high posterior causal probability identified in ref. ^28^. In **e** and **f**, error bars represent standard error (s.e.). Source data

The largest density of conditionally independent associations was observed on chromosome 15 near *ACAN*, a gene mutated in short stature and skeletal dysplasia syndromes, where 25 GWS SNPs co-localize within 100 kb of one another (Fig. 2 and Supplementary Fig. 17). We show in Supplementary Note 3 and Extended Data Fig. 5a–d, using haplotype- and simulation-based analyses, that a multiplicity of independent causal variants is the most likely explanation of this observation. We also found that signal density is partially explained by the presence of a recently identified^21,22^ height-associated variable-number tandem repeat (VNTR) polymorphism at this locus (Supplementary Note 3). In fact, the 25 independent GWS SNPs clustered within 100 kb of rs4932198 explain more than 40% of the VNTR length variation in multiple ancestries (Extended Data Fig. 5e), and an additional approximately 0.24% (*P* = 8.7 × 10^−55^) of phenotypic variance in EUR above what is explained by the VNTR alone (Extended Data Fig. 5f). Altogether, our conclusion is consistent with previous evidence of multiple types of common variation influencing height through *ACAN* gene function, involving multiple enhancers^23^, missense variants^24^ and tandem repeat polymorphisms^21,22^.

## Variance explained by SNPs within identified loci

To quantify the proportion of height variance that is explained by GWS SNPs identified in our META_FE_, we stratified all HM3 SNPs into two groups: SNPs in the close vicinity of GWS SNPs, hereafter denoted GWS loci; and all remaining SNPs. We defined GWS loci as non-overlapping genomic segments that contain at least one GWS SNP, such that GWS SNPs in adjacent loci are more than 2 × 35 kb away from each other (that is, a 35-kb window on each side). We chose this size window because it was predicted that causal variants are located within 35 kb of GWS SNPs with a probability greater than 80% (ref. ^25^). Accordingly, we grouped the 12,111 GWS SNPs identified in our META_FE_ into 7,209 non-overlapping loci (Supplementary Table 12) with lengths ranging from 70 kb (for loci containing only one signal) to 711 kb (for loci containing up to 25 signals). The average length of GWS loci is around 90 kb (s.d. 46 kb). The cumulative length of GWS loci represents around 647 Mb, or about 21% of the genome (assuming a genome length of around 3,039 Mb)^26^.

**Fig. 3:**
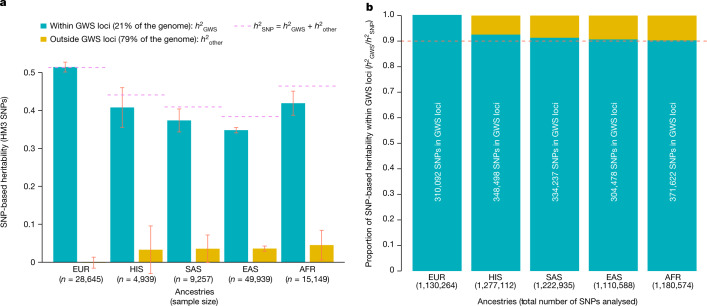
Variance of height explained by HM3 SNPs within GWS loci. **a**, Stratified SNP-based heritability (${h}_{{\rm{SNP}}}^{2}$) estimates obtained after partitioning the genome into SNPs within 35 kb of a GWS SNP ('GWS loci' label) versus SNPs that are more than 35 kb away from any GWS SNP. Analyses were performed in samples of five different ancestries or ethnic groups: European (EUR: meta-analysis of UK Biobank (UKB) + Lifelines study), African (AFR: meta-analysis of UKB + PAGE study), East Asian (EAS: meta-analysis of UKB + China Kadoorie Biobank), South Asian (SAS: UKB) and Hispanic (HIS: PAGE). Error bars represent standard errors. **b**, More than 90% of ${h}_{{\rm{SNP}}}^{2}$ in all ancestries is explained by SNPs within GWS loci identified in this study. The cumulative length of non-overlapping GWS loci is around 647 Mb; that is, around 21% of the genome, assuming a genome length of around 3,039 Mb (ref. ^26^). The proportion of HM3 SNPs in GWS loci is around 27%. Source data

To estimate the fraction of heritability that is explained by common variants within the 21% of the genome overlapping GWS loci, we calculated two genomic relationship matrices (GRMs)—one for SNPs within these loci and one for SNPs outside these loci—and then used both matrices to estimate a stratified SNP-based heritability (${h}_{{\rm{SNP}}}^{2}$) of height in eight independent samples of all five population groups represented in our META_FE_ (Fig. 3 and Methods). Altogether, our stratified estimation of SNP-based heritability shows that SNPs within these 7,209 GWS loci explain around 100% of ${h}_{{\rm{SNP}}}^{2}$ in EUR and more than 90% of ${h}_{{\rm{SNP}}}^{2}$ across all non-EUR groups, despite being drawn from less than 21% of the genome (Fig. 3). We also varied the window size used to define GWS loci and found that 35 kb was the smallest window size for which this level of saturation of SNP-based heritability could be achieved (Supplementary Fig. 18).

**Extended Data Fig. 6:**
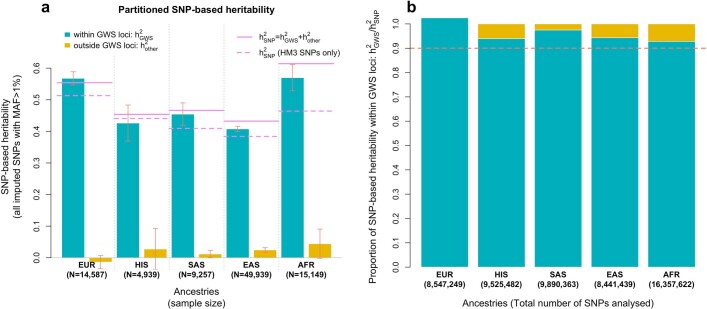
Variance of height explained by common SNPs within 35 kb of GWS SNPs. Stratified SNP-based heritability (${h}_{{\rm{SNP}}}^{2}$) estimates were obtained from a partition of the genome into two sets of 1000 Genomes imputed SNPs with a minor allele frequency (MAF) >1%: (1) SNPs within +/− 35 kb of GWS (GWS loci) vs. all other SNPs. Analyses were performed in samples of five different ancestry groups: European (EUR; UK Biobank only), African (AFR), East Asian (EAS) and South Asian (SAS) as described in the legend of Fig. 3. Estimates from stratified analyses were compared with SNP-based heritability estimates obtained from analysing HM3 SNPs only (dotted horizontal violet bar). Source data

To further assess the robustness of this key result, we tested whether the 7,209 height-associated GWS loci are systematically enriched for trait heritability. We chose body-mass index (BMI) as a control trait, given its small genetic correlation with height (*r*_g_ = −0.1, ref. ^27^) and found no significant enrichment of SNP-based heritability for BMI within height-associated GWS loci (Supplementary Fig. 19). Furthermore, we repeated our analysis using a random set of SNPs matched with the 12,111 height-associated GWS SNPs on EUR MAF and LD scores. We found that this control set of SNPs explained only around 27% of ${h}_{{\rm{SNP}}}^{2}$ for height, consistent with the proportion of SNPs within the loci defined by this random set of SNPs (Supplementary Figs. 18 and 19). Finally, we extended our stratified estimation of SNP-based heritability to all well-imputed common SNPs (that is, beyond the HM3 panel) and found, consistently across population groups, that although more genetic variance can be explained by common SNPs that are not included in the HM3 panel, all information remains concentrated within these 7,209 GWS loci (Extended Data Fig. 6). Thus, with this large GWAS, nearly all of the variability in height that is attributable to common genetic variants can be mapped to regions comprising around 21% of the genome. Further work is required in cohorts of non-European ancestries to map the remaining 5–10% of the SNP-based heritability that is not captured within those regions.

## Out-of-sample prediction accuracy

We quantified the accuracy of multiple polygenic scores (PGSs) for height on the basis of GWS SNPs (hereafter referred to as PGS_GWS_) and on the basis of all HM3 SNPs (hereafter referred to as PGS_HM3_). PGS_GWS_ were calculated using joint SNP effects from COJO, and PGS_HM3_ using joint effects calculated using the SBayesC method^28^ (Methods). We denote ${R}_{{\rm{GWS}}}^{2}$ and ${R}_{{\rm{HM}}3}^{2}$ as the prediction accuracy of PGS_GWS_ and PGS_HM3_, respectively. For conciseness, we also use the abbreviations PGS_GWS-X_ and PGS_HM3-X_ (and ${R}_{{\rm{GWS}}-{\rm{X}}}^{2}$ and ${R}_{{\rm{HM}}3-{\rm{X}}}^{2}$) to specify which GWAS meta-analysis each PGS (and corresponding prediction accuracy) was trained from. For example, PGS_GWS-METAFE_ refers to PGSs based on 12,111 GWS SNPs identified from our META_FE_.

**Fig. 4:**
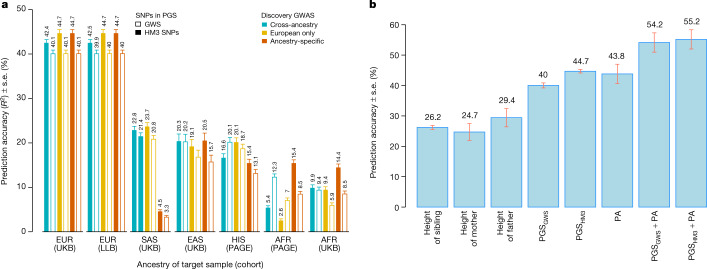
Accuracy of PGSs within families and across ancestries. Prediction accuracy (*R*^2^) was measured as the squared correlation between PGS and actual height adjusted for age, sex and 10 genetic principal components. **a**, Accuracy of PGSs assessed in participants of five different ancestry groups: European (EUR) from the UKB (*n* = 14,587) and the Lifelines Biobank (*n* = 14,058); South Asian (SAS; *n* = 9,257) from UKB; East Asian (EAS; *n* = 2,246) from UKB; Hispanic (HIS; *n* = 5,798) from the PAGE study; and admixed African (AFR) from UKB (*n* = 6,911) and PAGE (*n* = 8,238). PGSs used for prediction, in **a**, are based on GWS SNPs or around 1.1 million HM3 SNPs. When using all HapMap 3 SNPs, SNP effects were calculated using the SBayesC method (Methods), whereas PGSs based on GWS SNPs used joint SNP effects estimated using the COJO method (Methods). Both SBayesC and COJO were applied to (1) our cross-ancestry meta-analysis (turquoise bar); (2) our EUR meta-analysis (yellow bar); and (3) each ancestry-specific meta-analysis (red bar). **b**, Squared correlation of height between EUR participants in UKB and their first-degree relatives, and the accuracy of a predictor combining PGS (denoted PGS_GWS_, as based on GWS SNPs) and familial information. The accuracies of PGS_GWS_ and PGS_HM3_ shown in **b** are the average of the respective accuracies of these PGSs in EUR participants from UKB and the Lifelines Biobank as shown in **a**. Sibling correlation was calculated in 17,492 independent EUR sibling pairs from the UKB and parent–offspring correlations in 981 EUR unrelated trios (that is, two parents and one child) from the UKB. PA, parental average. Source data

**Extended Data Fig. 7:**
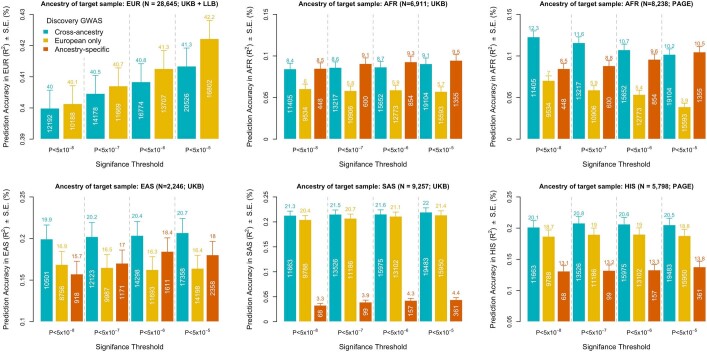
Accuracy of PGSs derived from joint effects of SNPs ascertained at various significance thresholds. The six panels show on their y-axes the prediction accuracy (R^2^) of multiple PGS across five target samples. The ancestry group and size of each target sample is indicated in the panel title. The top-left panel shows the averaged prediction accuracy in two European ancestry (EUR) target samples from the UK Biobank (UKB) and Lifelines Biobank (LLB). The other panels show prediction accuracies in individual target samples of African ancestry (AFR) from UKB and the PAGE study, East Asian ancestry (EAS) and South Asian ancestry (SAS) ancestry from the UKB and Hispanic ethnicity from the PAGE study. Each panel is divided in four columns representing the four significance levels used to ascertain SNPs using the GCTA-COJO algorithm. GCTA-COJO was applied to each ancestry-group specific GWAS meta-analysis with an ancestry-match linkage disequilibrium (LD) reference. We used genotypes from 50,000 (vs 350,000 for results reported in the main text) unrelated EUR participants as LD reference to run GCTA-COJO on the EUR- and the cross-ancestry GWAS meta-analysis. For the other ancestry groups, we used genotypes from 10,636 AFR individuals, 5,875 EAS individuals, 4,883 HIS individuals and 9,448 SAS individuals as LD reference (as described in Methods). Error bars are standard error (s.e.). The number of SNPs used in each PGS is indicated (in white) within each bar. Source data

We first present results from PGS_GWS_ across different ancestry groups. PGS_GWS-METAFE_ yielded prediction accuracies greater than or equal to that of all other PGS_GWS_ (Fig. 4a), partly reflecting sample size differences between ancestry-specific GWASs and also consistent with previous studies^29^. PGS_GWS-EUR_ (based on 9,863 SNPs) was the second best of all PGS_GWS_ across ancestry groups except in AFR. Indeed, PGS_GWS-AFR_ (based on 453 SNPs) yielded an accuracy of 8.5% (s.e. 0.6%) in AFR individuals from UKB and PAGE; that is, significantly larger than the 5.9% (s.e. 0.6%) and 7.0% (s.e. 0.6%) achieved by PGS_GWS-EUR_ in these two samples, respectively (Fig. 4a). PGS_GWS-METAFE_ was the best of all PGS_GWS_ in AFR participants with an accuracy ${R}_{{\rm{GWS}}-{\rm{METAFE}}}^{2}$ = (12.3% + 9.9%)/2 = 10.8% (s.e. 0.5%) on average between UKB and PAGE (Fig. 4a). Across ancestry groups, the highest accuracy of PGS_GWS-METAFE_ was observed in EUR participants (${R}_{{\rm{GWS}}-{\rm{METAFE}}}^{2}$~40%; s.e. 0.6%) and the lowest in AFR participants from the UKB (${R}_{{\rm{GWS}}-{\rm{METAFE}}}^{2}$ ≈ 9.4%; s.e. 0.7%). Note that the difference in ${R}_{{\rm{GWS}}-{\rm{METAFE}}}^{2}$ between the EUR and AFR ancestry cohorts is expected because of the over-representation of EUR in our META_FE_, and consistent with a relative accuracy (${R}_{{\rm{GWS}}-{\rm{METAFE}}}^{2}$ in AFR)/($\,{R}_{{\rm{GWS}}-{\rm{METAFE}}}^{2}$ in EUR) of around 25% that was previously reported^30^. We extended analyses of PGS_GWS_ to PGS based on SNPs identified with COJO at lower significance thresholds (Extended Data Fig. 7). As in previous studies^3,20^, the inclusion of sub-significant SNPs increased the accuracy of ancestry-specific PGSs. However, lowering the significance thresholds in our META_FE_ mostly improved accuracy in EUR (from 40% to 42%), whereas it slightly decreased the accuracy in AFR.

Overall, ancestry-specific PGS_HM3_ consistently outperform their corresponding PGS_GWS_ in most ancestry-groups. However, PGS_HM3_ was sometimes less transferable across ancestry groups than PGS_GWS_, in particular in AFR and HIS individuals from PAGE. In EUR, PGS_HM3_ reaches an accuracy of 44.7% (s.e. 0.6%), which is higher than previously published SNP-based predictors of height derived from individual-level data^31–33^ and from GWAS summary statistics^28,34,35^ across various experimental designs (different SNP sets, different sample sizes and so on). Finally, the largest improvement of PGS_HM3_ over PGS_GWS_ was observed in AFR individuals from the PAGE study (${R}_{{\rm{GWS}}-{\rm{AFR}}}^{2}$ = 8.5% versus ${R}_{{\rm{HM}}3}^{2}$ = 15.4%; Fig. 4a) and the UKB (${R}_{{\rm{GWS}}-{\rm{AFR}}}^{2}$ = 8.5% versus ${R}_{{\rm{HM}}3}^{2}$ = 14.4%; Fig. 4a).

Furthermore, we sought to evaluate the prediction accuracy of PGSs relative to that of familial information as well as the potential improvement in accuracy gained from combining both sources of information. We analysed 981 unrelated EUR trios (that is, two parents and one child) and 17,492 independent EUR sibling pairs from the UKB, who were excluded from our META_FE_. We found that height of any first-degree relative yields a prediction accuracy between 25% and 30% (Fig. 4b). Moreover, the accuracy of the parental average is around 43.8% (s.e. 3.2%), which is lower than yet not significantly different from the accuracy of PGS_HM3-EUR_ in EUR. In addition, we found that a linear combination of the average height of parents and of the child’s PGS yields an accuracy of 54.2% (s.e. 3.2%) with PGS_GWS-EUR_ and 55.2% (s.e. 3.2%) with PGS_HM3-EUR_. This observation reflects the fact that PGSs can explain within-family differences between siblings, whereas average parental height cannot. To show this empirically, we estimate that our PGSs based on GWS SNPs explain around 33% (s.e. 0.7%) of height variance between siblings (Methods). Finally, we show that the optimal weighting between parental average and PGS can be predicted theoretically as a function of the prediction accuracy of the PGS, the full narrow sense heritability and the phenotypic correlation between spouses (Supplementary Note 4 and Supplementary Fig. 20).

In summary, the estimation of variance explained and prediction analyses in samples with European ancestry show that the set of 12,111 GWS SNPs accounts for nearly all of ${h}_{{\rm{SNP}}}^{2}$, and that combining SNP-based PGS with family history significantly improves prediction accuracy. By contrast, both estimation and prediction results show clear attenuation in samples with non-European ancestry, consistent with previous studies^30,36–38^.

## GWAS discoveries, sample size and ancestry diversity

Our large study offers the opportunity to quantify empirically how much increasing GWAS sample sizes and ancestry diversity affects the discovery of variants, genes and biological pathways. To address this question, we re-analysed three previously published GWASs of height^3,19,20^ and also down-sampled our meta-analysis into four subsets (including our EUR and META_FE_ GWASs). Altogether, we analysed seven GWASs with a sample size increasing from around 0.13 million up to around 5.3 million individuals (Table 2).

**Table 2:** Table 2 Overview of five European-ancestry GWASs re-analysed in our study to quantify the relationship between sample size and discovery

Down-sampled GWAS	Max *n* (mean *n*)	Number of GWS COJO SNPs	Percentage of the genome covered by GWS loci (35 kb) (%)
Lango Allen et al. (2010)^19^^a^	130,010 (128,942)	240	0.5
Wood et al. (2014)^20^	241,724 (239,227)	633	1.4
Yengo et al. (2018)^3^	695,648 (688,927)	2,794	5.8
GIANT-EUR (no 23andMe)	1,632,839 (1,502,499)	4,867	9.7
23andMe-EUR	2,502,262 (2,498,336)	7,020	13.6

Summary statistics from the three published GWASs were imputed using the ImpG-Summary software to maximize the coverage of HM3 SNPs (Methods). GWS loci are defined as in the legend of Table 1.

^a^Summary statistics from the Lango Allen et al. study^19^, initially over-corrected for population stratification using a double genomic control correction, were re-inflated such that the LD score regression intercept estimated from re-inflated test statistics equals 1.

For each GWAS, we quantified eight metrics grouped into four variant- and locus-based metrics (number of GWS SNPs; number of GWS loci; prediction accuracy (${R}_{{\rm{GWS}}}^{2}$) of PGS based on GWS SNPs; and proportion of the genome covered by GWS loci), a functional-annotation-based metric (enrichment statistics from stratified LDSC^39,40^), two gene-based metrics (number of genes prioritized by summary-data-based Mendelian randomization^41^ (SMR; Methods) and proximity of variants with OMIM genes) and a gene-set-based metric (enrichment within clusters of gene sets or pathways). Overall, we found different patterns for the relationship between those metrics and GWAS sample size and ancestry composition, consistent with varying degrees of saturation achieved at different sample sizes.

**Extended Data Fig. 8:**
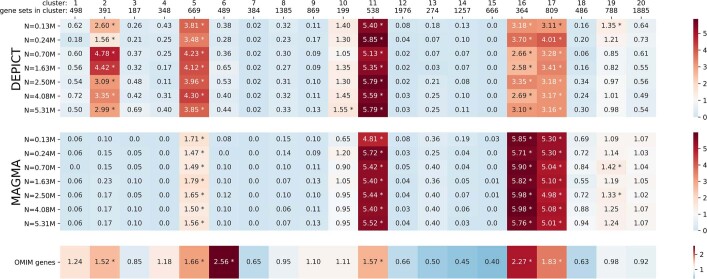
Enrichment of height-associated genes identified at various GWAS sample sizes within 20 clusters of gene sets representing broad categories of biological pathways. Gene-set enrichment was performed with MAGMA and DEPICT across seven GWAS with increasing sample sizes. Samples used (Lango Allen et al. (2010), *n* = 0.13M; Wood et al. (2014), *n* = 0.24M; Yengo et al. (2018), *n* = 0.7M; GIANT-EUR (no 23andMe), *n* = 1.63M; 23andMe-EUR, *n* = 2.5M; European-ancestry meta-analysis, *n* = 4.08M; and cross-ancestry meta-analysis, *n* = 5.31M) are described in Tables 1–2. The degree of enrichment of gene sets (MAGMA, DEPICT) of known skeletal growth disorder genes catalogued in the Online Mendelian Inheritance in Man (OMIM) database among 20 clusters of gene sets (see Methods section in Supplementary Note 5) is indicated by the blue-red colour scale. Enrichment for MAGMA and DEPICT was defined to be the number of prioritized gene sets (top 10% of gene sets) in each cluster divided by the 10% of the number of gene sets in the cluster. Enrichment for OMIM was defined to be the number of OMIM genes in a gene set (Z > 1.96) divided by the size of the gene set divided by the proportion of all genes in OMIM, then averaged across the cluster. Significant enrichment (compared to shuffled prioritization of gene sets or genes) is marked with *. Source data

**Extended Data Fig. 9:**
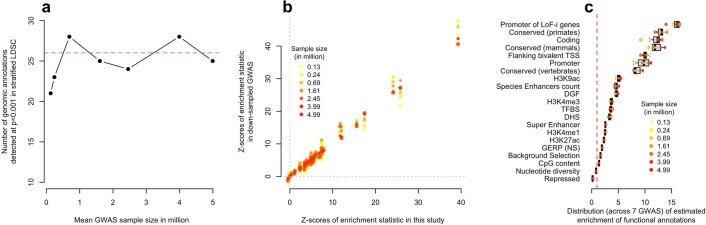
Annotation-level saturation of GWAS discoveries as a function of sample size. Increase in sample size from ~4 million to ~5 million is achieved by including ~1 million participants of non-European ancestry. **a**, Number of annotations showing a significant heritability enrichment as function the function of the sample size of the GWAS used to estimate these enrichment. Heritability enrichment was detected using a stratified LD score regression (LDSC) analysis of 97 genomic annotations included in the “baseline + LD” model from Gazal et al. **b**, Correlation between Z-scores measuring the statistical significance of heritability enrichments of 97 annotations (each dot is an annotation) in our largest GWAS (x-axis) as compared to down-sampled GWAS (y-axis). Sample size is denoted by the colour-code. **c**, Distribution of estimated enrichment statistics for 21 annotations found significantly enriched (*P* < 0.05/97) in at least 6 of the 7 GWAS analysed here. LoF-i genes: Loss of function intolerant genes; TSS: Transcription Start Sites; DGF: Digital genomic footprint; TFBS: Transcription Factor Binding Sites; DHS: DNAse I hypersensitive sites; GERP (NS): GERP++ score (number of substitutions). Source data

We observed the strongest saturation for the gene-set and functional-annotation metrics, which capture how well general biological functions can be inferred from GWAS results using currently available computational methods. Using two popular gene-set prioritization methods (DEPICT^42^ and MAGMA^43^), we found that the same broad clusters of related gene sets (including most of the clusters enriched for OMIM genes) are prioritized at all GWAS sample sizes (Supplementary Fig. 21, Extended Data Fig. 8, Supplementary Tables 13–15 and Supplementary Note 5). Similarly, stratified LDSC estimates of heritability enrichment within 97 functional annotations also remain stable across the range of sample sizes (Extended Data Fig. 9). Overall, we found no significant improvement for all these higher-level metrics from adding non-EUR samples to our analyses. The latter observation is consistent with other analyses showing that GWASs expectedly implicate similar biology across major ancestral groups (Supplementary Note 5 and Supplementary Fig. 22).

For the gene-level metric, the excess in the number of OMIM genes that are proximate to a GWS SNP (compared with matched sets of random genes) plateaus at sample sizes of larger than 1.5 million, whereas the relative enrichment of GWS SNPs near OMIM genes first decreases with sample size, then plateaus when *n* is greater than 1.5 million (Supplementary Fig. 23a–c). Notably, the decrease observed for *n* values of less than 1.5 million reflects the preferential localization of larger effect variants (those identified with smaller sample sizes) closer to OMIM genes (Supplementary Fig. 23d) and, conversely, that more recently identified variants with smaller effects tend to localize further away from OMIM genes (Supplementary Fig. 23e). We also investigated the number of genes prioritized using SMR (hereafter referred to as SMR genes; Methods) using expression quantitative trait loci (eQTLs) as genetic instruments (Supplementary Table 16) as an alternative gene-level metric and found it to saturate for *n* values greater than 4 million (Supplementary Fig. 23f). Note that saturation of SMR genes is partly affected by the statistical power of current eQTL studies, which do not always survey biologically relevant tissues and cell types for height. Therefore, we can expect more genes to be prioritized when integrating GWAS summary statistics from this study with those from larger eQTL studies that may be available in the future and may involve more tissue types. Gene-level metrics were also not substantially affected by adding non-EUR samples, again consistent with broadly similar sets of genes affecting height across ancestries.

At the level of variants and genomic regions, we saw a steady and almost linear increase in the number of GWS SNPs as a function of sample size, as previously reported^44^. However, given that newly identified variants tend to cluster near ones identified at smaller sample sizes, we also saw a saturation in the number of loci identified for *n* values greater than 2.5 million, where the upward trend starts to weaken (Supplementary Fig. 24a). We found a similar pattern for the percentage of the genome covered by GWS loci, with the degree of saturation varying as a function of the window size used to define loci (Supplementary Fig. 24b). The observed saturation in PGS prediction accuracy (both within ancestry—that is, in EUR—and multi-ancestry) was more noticeable than that of the number and genomic coverage of GWS loci. In fact, increasing the sample size from 2.5 million to 4 million by adding another 1.5 million EUR samples increased the number of GWS SNPs from 7,020 to 9,863—that is, an increase of around 1.4-fold ((9,863 − 7,020)/7,020)—but the absolute increase in prediction accuracy is less than 2.7%. This improvement is mainly observed in EUR but remains lower than 1.3% in individuals of the EAS and AFR ancestry groups. However, adding another approximately 1 million participants of non-EUR improves the multi-ancestry prediction accuracy by more than 3.4% (Supplementary Fig. 24c), highlighting the value of including non-EUR populations.

Altogether, these analyses show that increasing the GWAS sample size not only increases the prediction accuracy, but also sheds more light on the genomic distribution of causal variants and, at all but the largest sample sizes, the genes proximal to these variants. By contrast, enrichment of higher-level, broadly defined biological categories such as gene sets and pathways and functional annotations can be identified using relatively small sample sizes (*n* ≈ 0.25 million for height). Of note, we confirm that increased genetic diversity in GWAS discovery samples significantly improves the prediction accuracy of PGSs in under-represented ancestries.

## Discussion

By conducting one of the largest GWASs so far in 5.4 million individuals, with a primary focus on common genetic variation, we have provided insights into the genetic architecture of height—including a saturated genomic map of 12,111 genetic associations for height. Consistent with previous studies^19,20^, we have shown that signal density of associations (known and novel) is not randomly distributed across the genome; rather, associated variants are more likely to be detected around genes that have been previously associated with Mendelian disorders of growth. Furthermore, we observed a strong genetic overlap of association across cohorts with various ancestries. Effect estimates of associated SNPs are moderately to highly correlated (minimum = 0.64; maximum = 0.99), suggesting even larger correlations of effect sizes of underlying causal variants^13^. Moreover, although there are significant differences in power to detect an association between cohorts with European and non-European ancestries, most genetic associations for height observed in populations with non-European ancestry lie in close proximity and in linkage disequilibrium to associations identified within populations of European ancestry.

**Extended Data Fig. 10:**
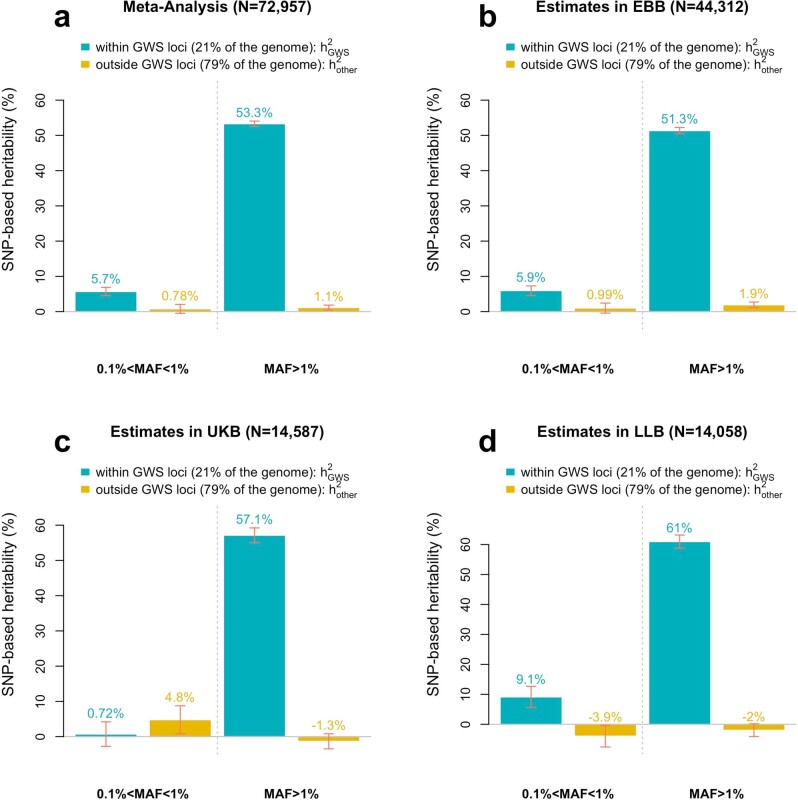
Partitioning of low-frequency SNP-based heritability within GWS loci. Panels **b**–**d** represent partitioned SNP-based heritability estimates from three samples (EBB: Estonian Biobank; UKB: UK Biobank; LLB: Lifelines Biobank) of unrelated European ancestry individuals independent of our discovery GWAS. **a**, Partitioned SNP-based heritability estimates obtained from an inverse-variance weighted meta-analysis of estimates shown in **b**–**d**. SNPs were partitioned into four classes according to their minor allele frequency (MAF: 0.1% < MAF < 1% vs. MAF > 1%) and their position within versus outside GWS loci. The SNP-based heritability contributed by SNPs within GWS loci is denoted ${h}_{{\rm{GWS}}}^{2}$, and that contributed by SNPs outside these loci is denoted ${h}_{{\rm{other}}}^{2}$. These results are further discussed in Supplementary Note 6. Source data

By increasing our experimental sample size to more than seven times that of previous studies, we have explained up to 40% of the inter-individual variation in height in independent European-ancestry samples using GWS SNPs alone, and more than 90% of ${h}_{{\rm{SNP}}}^{2}$ across diverse populations when incorporating all common SNPs within 35 kb of GWS SNPs. This result highlights that future investigations of common (MAF > 1%) genetic variation associated with height in many ancestries will be most likely to detect signals within the 7,209 GWS loci that we have identified in the present study. A question for the future is whether rare genetic variants associated with height are also concentrated within the same loci. We provide suggestive evidence supporting this hypothesis from analysing imputed SNPs with 0.1% < MAF < 1% (Supplementary Note 6, Extended Data Fig. 10 and Supplementary Fig. 25). Our results are consistent with findings from a previous study^45^, which showed across 492 traits a strong colocalization between common and rare coding variants associated with the same trait. Nevertheless, our conclusions remain limited by the relatively low performances of imputation in this MAF regime^46,47^. Therefore, large samples with whole-genome sequences will be required to robustly address this question. Such datasets are increasingly becoming available^48–50^. Separately, previous studies have reported a significant enrichment of height heritability near genes as compared to inter-genic regions (that is, >50 kb away from the start or stop genomic position of genes)^51^. Our findings are consistent with but not reducible to that observation, given that up to 31% of GWS SNPs identified in this study lie more than 50 kb away from any gene.

Our study provides a powerful genetic predictor of height based on 12,111 GWS SNPs, for which accuracy reaches around 40% (that is, 80% of ${h}_{{\rm{SNP}}}^{2}$) in individuals of European ancestries and up to around 10% in individuals of predominantly African ancestries. Notably, we show using a previously developed method^38^ that LD and MAF differences between European and African ancestries can explain up to around 84% (s.e. 1.5%) of the loss of prediction accuracy between these populations (Methods), with the remaining loss being presumably explained by differences in heritability between populations and/or differences in effect sizes across populations (for example, owing to gene-by-gene or gene-by-environment interactions). This observation is consistent with common causal variants for height being largely shared across ancestries. Therefore, we anticipate that fine-mapping of GWS loci identified in this study, ideally using methods that can accommodate dense sets of signals and large populations with African ancestries, would substantially improve the accuracy of a derived height PGS for populations of non-European ancestry. Our study has a large number of participants with African ancestries as compared with previous efforts. However, we emphasize that further increasing the size of GWASs in populations of non-European ancestry, including those with diverse African ancestries, is essential to bridge the gap in prediction accuracy—particularly as most studies only partially capture the wide range of ancestral diversity both within Africa and globally. Such increased sample sizes would help to identify potential ancestry-specific causal variants, to facilitate ancestry-specific fine-mapping and to inform gene–environment and gene–ancestry interactions. Another important finding of our study is to show how individual PGS can be optimally combined with familial information and thereby improve the overall accuracy of height prediction to above 54% in populations of European ancestry.

Although large sample sizes are needed to pinpoint the variants responsible for the heritability of height (and larger samples in multiple ancestries will probably be required to map these at finer scale), the prioritization of relevant genes and gene sets is feasible at smaller sample sizes than that required to account for the common variant heritability. Thus, the sample sizes required for saturation of GWAS are smaller for identifying enriched gene sets, with the identification of genes implicated as potentially causal and mapping of genomic regions containing associated variants requiring successively larger sample sizes. Furthermore, unlike prediction accuracy, prioritization of genes that are likely to be causal and even mapping of associated regions is consistent across ancestries, reflecting the expected similarity in the biological architecture of human height across populations. Recent studies using UKB data predicted that GWAS sample sizes of just over 3 million individuals are required to identify 6,000–7,000 GWS SNPs explaining more than 90% of the SNP-based heritability of height^52^. We showed empirically that these predictions are downwardly biased given that around 10,000 independent associations are, in fact, required to explain 80–90% of the SNP-based heritability of height in EUR individuals. Discrepancies between observed and predicted levels of saturation could be explained by several factors, such as (i) heterogeneity of SNP effects between cohorts and background ancestries, which may have reduced the statistical power of our study as compared to a homogenous sample like UKB; (ii) inconsistent definitions of GWS SNPs (using COJO in this study versus standard clumping in ref. ^52^); and, most importantly, (iii) misspecification of the SNP-effects distribution assumed to make these predictions. Nevertheless, if these predictions reflect proportional levels of saturation between traits, then we could expect that two- to tenfold larger samples would be required for GWASs of inflammatory bowel disease (×2, that is, *n* = 10 million), schizophrenia (×7; *n* = 35 million) or BMI (×10; *n* = 50 million) to reach a similar saturation of 80–90% of SNP-based heritability.

Our study has a number of limitations. First, we focused on SNPs from the HM3 panel, which only partially capture common genetic variation. However, although a significant fraction of height variance can be explained by common SNPs outside the HM3 SNPs panel, we showed that the extra information (also referred to as ‘hidden heritability’) remains concentrated within GWS loci identified in our HM3-SNP-based analyses (Extended Data Fig. 6). This result underlines the widespread allelic heterogeneity at height-associated loci. Another limitation of our study is that we determined conditional associations using a EUR LD reference (*n* ≈ 350,000), which is sub-optimal given that around 24% of our discovery sample is of non-European ancestry. We emphasize that no analytical tool with an adequately large multi-ancestry reference panel is at present available to properly address how to identify conditionally independent associations in a multi-ancestry study. Fine-mapping of variants remains a particular challenge when attempted across ancestries in loci containing multiple signals (as is often the case for height).A third limitation of our study is our inability to perform well-powered replication analyses of genetic associations specific to populations with non-European ancestries, owing to the current limited availability of such data. Finally, as with all GWASs, definitive identification of effector genes and the mechanisms by which genes and variants influence phenotype remains a key bottleneck. Therefore, progress towards identifying causal genes from GWAS of height may be achieved by a combination of increasingly large whole-exome sequencing studies, allowing straightforward SNP-to-gene mapping^45^, the use of relevant complementary data (for example, context-specific eQTLs in relevant tissues and cell types) and the development of computational methods that can integrate these data.

In summary, our study has been able to show empirically that the combined additive effects of tens of thousands of individual variants, detectable with a large enough experimental sample size, can explain substantial variation in a human phenotype. For human height, we show that studies of the order of around 5 million participants of various ancestries provide enough power to map more than 90% (around 100% in populations of European ancestry) of genetic variance explained by common SNPs down to around 21% of the genome. Mapping the missing 5–10% of SNP-based heritability not accounted for in the four non-European ancestries studied here will require additional and directed efforts in the future.

Height has been used as a model trait for the study of human polygenic traits, including common diseases, because of its high heritability and relative ease of measurement, which enable large sample sizes and increased power. Conclusions about the genetic architecture, sample size requirements for additional GWAS discovery and scope for polygenic prediction that were initially made for height have by-and-large agreed with those for common disease. If the results from this study can also be extrapolated to disease, this would suggest that substantially increased sample sizes could largely resolve the heritability attributed to common variation to a finite set of SNPs (and small genomic regions). These variants and regions would implicate a particular subset of genes, regulatory elements and pathways that would be most relevant to address questions of function, mechanism and therapeutic intervention.

## Methods

A summary of the methods, together with a full description of genome-wide association analyses and follow-up analyses is described below. Written informed consent was obtained from every participant in each study, and the study was approved by relevant ethics committees (Supplementary Table 1).

### Quality control checks of individual studies

All study files were checked for quality using the software EasyQC^53^ that was adapted to the format from RVTESTS (versions listed in Supplementary Table 2)^54^. The checks performed included allele frequency differences with ancestry-specific reference panels, total number of markers, total number of markers not present in the reference panels, imputation quality, genomic inflation factor and trait transformation. We excluded two studies that did not pass our quality checks in the data.

### GWAS meta-analysis

We first performed ancestry-group-specific GWAS meta-analyses of 173 studies of EUR, 56 studies of EAS, 29 studies of AFR, 11 studies of HIS and 12 studies of SAS. Meta-analyses within ancestry groups were performed as described before^19,20^ using a modified version of RAREMETAL^55^ (v.4.15.1), which accounts for multi-allelic variants in the data. Study-specific GWASs are described in Supplementary Tables 1–3. Details about imputation procedures implemented by each study are also given in Supplementary Table 2. We kept in our analyses SNPs with an imputation accuracy (${r}_{{\rm{INFO}}}^{2}$) > 0.3, Hardy–Weinberg Equilibrium (HWE) *P* value (*P*_HWE_) > 10^−8^ and a minor allele count (MAC) > 5 in each study. Next, we performed a fixed-effect inverse variance weighted meta-analysis of summary statistics from all five ancestry groups GWAS meta-analysis using a custom R script using the R package meta (see ‘URLs’ section).

### Hold-out sample from the UK Biobank

We excluded 56,477 UK Biobank (UKB) participants from our discovery GWAS for following analyses including quantification of population stratification. More precisely, our hold-out EUR sample consists of 17,942 sibling pairs and 981 trios (two parents and one child) plus all UKB participants with an estimated genetic relationship larger than 0.05 with our set of sibling pairs and trios. We identified 14,587 individuals among these 56,477 UKB participants who were unrelated (unrelatedness was determined as when the genetic relationship coefficient estimated from HM3 SNPs  was  lower than 0.05) to each other and used their data to quantify the variance explained by SNPs within GWS loci (described below) and the prediction accuracy of PGSs.

### COJO analyses

 We performed COJO analyses of each of the five ancestry group-specific GWAS meta-analyses using the software GCTA (version v.1.93)^6,7^. We used default parameters for all ancestry groups except in AFR and HIS, for which we found that default parameters could yield biased estimates of joint SNP effects because of long-range LD. This choice is discussed in Supplementary Note 1. The GCTA-COJO method implements a stepwise model selection that aims at retaining a set of SNPs the joint effects of which reach genome-wide significance, defined in this study as *P* < 5 × 10^−8^. In addition to GWAS summary statistics, COJO analyses also require genotypes from an ancestry-matched sample that is used as a LD reference. For all sets of genotypes used as LD reference panels, we selected HM3 SNPs with ${r}_{{\rm{INFO}}}^{2}$ > 0.3 and *P*_HWE_ > 10^−6^. For EUR, we used genotypes at 1,318,293 HM3 SNPs (MAC > 5) from 348,501 unrelated EUR participants in the UKB as our LD reference. For EAS, we used genotypes at 1,034,263 quality-controlled (MAF > 1%, SNP missingness < 5%) HM3 SNPs from a merged panel of *n* = 5,875 unrelated participants from the UKB (*n* = 2,257) and Genetic Epidemiology Research on Aging (GERA; *n* = 3,618). Data from the GERA study were obtained from the database of Genotypes and Phenotypes (dbGaP; accession number: phs000788.v2.p3.c1) under project 15096. For SAS, we used genotypes at 1,222,935 HM3 SNPs (MAC > 5; SNP missingness < 5%) from 9,448 unrelated individuals. For AFR, we used genotypes at 1,007,949 quality-controlled (MAF > 1%, SNP missingness < 5%) HM3 SNPs from a merged panel of 15,847 participants from the Women’s Health Initiative (WHI; *n* = 7,480), and the National Heart, Lung, and Blood Institute’s Candidate Gene Association Resource (CARe^56^, *n* = 8,367). Both WHI and CARe datasets were obtained from dbGaP (accession numbers: phs000386 for WHI; CARe including phs000557.v4.p1, phs000286.v5.p1, phs000613.v1.p2, phs000284.v2.p1, phs000283.v7.p3 for ARIC, JHS, CARDIA, CFS and MESA cohorts) and processed following the protocol provided by the dbGaP data submitters. After excluding samples with more than 10% missing values and retaining only unrelated individuals, our final LD reference included data from *n* = 10,636 unrelated AFR individuals. For HIS, we used genotypes at 1,246,763 sequenced HM3 SNPs (MAF > 1%) from *n* = 4,883 unrelated samples from the Hispanic Community Health Study/Study of Latinos (HCHS/SOL; dbGaP accession number: phs001395.v2.p1) cohorts. Finally, we performed a COJO analysis of the combined meta-analysis of all ancestries (referred to as META_FE_ in the main text) using 348,501 unrelated EUR participants in the UKB as the reference panel.

To assess whether SNPs detected in non-EUR were independent of signals detected in EUR, we performed another COJO analysis of ancestry groups GWAS by fitting jointly SNPs detected in EUR with those detected in each of the non-EUR GWAS meta-analyses. For each non-EUR GWAS, we performed a single-step COJO analysis only including SNPs identified in that non-EUR GWAS and for which the LD squared correlation (${r}_{{\rm{LD}}}^{2}$) with any of the EUR signals (marginally or conditionally GWS) is lower than 0.8 in both EUR and corresponding non-EUR data. Single-step COJO analyses were performed using the --cojo-joint option of GCTA, which does not involve model selection and simply approximates a multivariate regression model in which all selected SNPs on a chromosome are fitted jointly. LD correlations used in these filters were estimated in ancestry-matched samples of the 1000 Genomes Project (1KGP; release 3). More specifically, LD was estimated in 661 AFR, 347 HIS (referred to with the AMR label in 1KGP), 504 EAS, 503 EUR and 489 SAS 1KGP participants. We used the same LD reference samples in these analyses as for our main discovery analysis described at the beginning of the section.

### *F*_ST_ calculation and (stratified) LD score regression

We used two statistics to evaluate whether an EUR LD reference could approximate well enough the LD structure in our trans-ancestry GWAS meta-analysis. The first statistic that we used is the Wright fixation index^57^, which measures allele frequency divergence between two populations. We used the Hudson’s estimator of *F*_ST_^58^ as previously recommended^59^ to compare allele frequencies from our META_FE_ with that from our EUR GWAS meta-analysis and an independent replication sample from the EBB. The other statistic that we used is the attenuation ratio statistic from the LD score regression methodology. These LD score regression analyses were performed using version 1.0 of the LDSC software and using LD scores calculated from EUR participants in the 1KGP (see ‘URLs’ section). Moreover, we performed a stratified LD score regression analysis to quantify the enrichment of height heritability in 97 genomic annotations curated and described previously^40^. as the baseline-LD model. Annotation-weighted LD scores used for those analyses were also calculated using data from 1KGP (see ‘URLs’ section).

### Density of GWS signal and enrichment near OMIM genes

We defined the density of independent signals around each GWS SNP as the number of other independent associations identified with COJO within a 100-kb window on both sides. Therefore, a SNP with no other associations within 100 kb has a density of 0, whereas a SNP colocalizing with 20 other GWS associations within 100 kb will have a density of 20. We quantified the standard error of the mean signal density across the genome using a leave-one-chromosome-out jackknife procedure. We then quantified the enrichment of 462 curated OMIM^18^ genes near GWS SNPs with a large signal density, by counting the number of OMIM genes within 100 kb of a GWS SNP, then comparing that number for SNPs with a density of 0 and those with a density of at least 1. The strength of the enrichment was measured using an odds ratio calculated from a 2×2 contingency table: 'presence/absence of an OMIM gene' versus 'density of 0 or larger than 0'. To assess the significance of the enrichment, we simulated the distribution of enrichment statistics for a random set of 462 length-matched genes. We used 22 length classes (<10 kb; between *i* × 10 kb and (*i* + 1) × 10 kb, with *i* = 1,…,9; between i × 100 kb and (*i* + 1) × 100 kb, with *i* = 1,…,10; between 1 Mb and 1.5 Mb; between 1.5 Mb and 2 Mb; and >2 Mb) to match OMIM genes with random genes. OMIM genes within a given length class were matched with the same number of non-OMIM genes present in the class. We sampled 1,000 random sets of genes and calculated for each them an enrichment statistic. Enrichment *P* value was calculated as the number of times enrichment statistics of random genes exceeded that of OMIM genes. The list of OMIM genes is provided in Supplementary Table 11.

### Genomic colocalization of GWS SNPs identified across ancestries

We assessed the genomic colocalization between 2,747 GWS SNPs identified in non-EUR (Supplementary Tables 5–8) and 9,863 GWS SNPs identified in EUR (Supplementary Table 4) by quantifying the proportion of EUR GWS SNPs identified within 100 kb of any non-EUR GWS SNP. We tested the statistical significance of this proportion by comparing it with the proportion of EUR GWS SNPs identified within 100 kb of random HM3 SNPs matched with non-EUR GWS SNPs on 24 binary functional annotations^39^.

These 24 annotations (for example, coding or conserved) are thoroughly described in a previous study^39^ and were downloaded from https://alkesgroup.broadinstitute.org/LDSCORE/baselineLD_v2.1_annots/.

Our matching strategy consists of three steps. First, we calibrated a statistical model to predict the probability for a given HM3 SNP to be GWS in any of our non-EUR GWAS meta-analyses as a function of their annotation. For that, we used a logistic regression of the non-EUR GWS status (1 = if the SNP is GWS in any of the non-EUR GWAS; 0 = otherwise) onto the 24 annotations as regressors. Second, we used that model to predict the probability to be GWS in non-EUR. Thirdly, we used the predicted probability to sample (with replacement) 1,000 random sets of 2,747 SNPs. Finally, we estimated the proportion of EUR GWS SNPs within 100 kb of SNPs in each sampled SNP set. We report in the main text the mean and s.d. over these 1,000 proportions.

To validate our matching strategy, we compared the mean value of each of these 24 annotations (for example, proportion of coding SNPs) between non-EUR GWS SNPs and each of the 1,000 random sets of SNPs, using a Fisher’s exact test. For each of the 24 annotations, both the mean and median *P* value were greater than 0.6 and the proportion of *P* values < 5% was less than 1%, suggesting no significant differences in the distribution of these 24 annotations between non-EUR GWS SNPs and matched SNPs.

### Replication analyses

To assess the replicability of our results, we tested whether the correlation *ρ*_b_ of estimated SNP effects between our discovery GWAS and our replication sample of 49,160 participants of the EBB was statistically different from 1. We used the estimator of *ρ*_b_ from a previous study^60^, which accounts for sampling errors in both discovery and replication samples. Standard errors were calculated using a leave-one-SNP-out jackknife procedure. We quantified the correlation of marginal and also that of joint SNP effects. Joint SNP effects in our replication sample were obtained by performing a single-step COJO analysis of GWAS summary statistics from our EBB sample, using the same LD reference as in the discovery GWAS. Correlation of SNP effects were calculated after correcting SNP effects for winner’s curse using a previously described method^12^. We provide the R scripts used to apply these corrections and estimate the correlation of SNP effects (see ‘URLs’ section). The expected proportion, *E*[*P*], of sign-consistent SNP effects between discovery and replication was calculated using the quadrant probability of a standard bivariate Gaussian distribution with correlation *E*[*ρ*_b_], denoting the expected correlation between estimated SNP effects in the discovery and replication sample:

1 $$E[P]=\frac{1}{2}+\frac{{\sin }^{-1}(E[{\rho }_{{\rm{b}}}t])}{\pi },$$

where sin^−1^ denotes the inverse of the sine function and *E*[*ρ*_b_] the expectation of the *ρ*_b_ statistic under the assumption that the true SNP effects are the same across discovery and replications cohorts. E[*ρ*_b_] was calculated as

2 $$E[\,{\rho }_{{\rm{b}}}]=\,\frac{{\sigma }_{{\rm{b}}}^{2}}{\sqrt{\left({\sigma }_{{\rm{b}}}^{2}\,+\,[1-{\sigma }_{{\rm{b}}}^{2}{h}_{{\rm{d}}}]/({N}_{{\rm{d}}}{h}_{{\rm{d}}})\,\right)\left({\sigma }_{{\rm{b}}}^{2}\,+\,[1-{\sigma }_{{\rm{b}}}^{2}{h}_{{\rm{r}}}]/({N}_{{\rm{r}}}{h}_{{\rm{r}}})\right)}},$$

where *N*_d_ and *N*_r_ denote the sizes of the discovery and replication samples, respectively; *h*_d_ and *h*_r_ the average heterozygosity under Hardy–Weinberg equilibrium (that is, 2 × MAF × (1 − MAF)) across GWS SNPs in the discovery and replication samples, respectively; and ${{\rm{\sigma }}}_{{\rm{b}}}^{2}$ the mean per-SNP variance explained by GWS SNPs, which we calculated (as per ref. ^60^.) as the sample variance of estimated SNP effects in the discovery sample minus the median squared standard error.

### Variance explained by GWS SNPs and loci

We estimated the variance explained by GWS SNPs using the genetic relationship-based restricted maximum likelihood (GREML) approach implemented in GCTA^1,7^. This approach involves two main steps: (i) calculation of genetic relationships matrices (GRM); and (ii) estimation of variance components corresponding to each of these matrices using a REML algorithm. We partitioned the genome in two sets containing GWS loci on the one hand and all other HM3 SNPs on the other hand. GWS loci were defined as non-overlapping genomic segments containing at least one GWS SNP and such that GWS SNPs in adjacent loci are more than 2 × 35 kb away from each other (that is, a 35-kb window on each side). We then calculated a GRM based on each set of SNPs and estimated jointly a variance explained by GWS alone and that explained by the rest of the genome. We performed these analyses in multiple samples independent of our discovery GWAS, which include participants of diverse ancestry. Details about the samples used for these analyses are provided below. We extended our analyses to also quantify the variance explained by GWS loci using alternative definitions based on a window size of 0 kb and 10 kb around GWS SNPs (Supplementary Figs. 18 and 19).

We also repeated our analyses using a random set of 12,111 SNPs matched with GWS SNPs on MAF and LD. Loci for these 12,111 random SNPs were defined similarly as for GWS loci. To match random SNPs with GWS SNPs on MAF and LD, we first created 28 MAF-LD classes of HM3 SNPs (7 MAF classes × 4 LD score classes). MAF classes were defined as <1%; between 1% and 5%; between 5% and 10%; between 10% and 20%; between 20% and 30%; between 30% and 40%; and between 40% and 50%. LD score classes were defined using quartiles of the HM3 LD score distribution. We next matched GWS SNPs in each of the 28 MAF-LD classes, with the same number of SNPs randomly sampled from that MAF-LD class.

### Prediction analyses

Height was first mean-centred and scaled to variance 1 within each sex. We quantified the prediction accuracy of height predictors as the difference between the variance explained by a linear regression model of sex-standardized height regressed on the height predictor, age, 20 genotypic principal components and study-specific covariates (full model) minus that explained by a reduced linear regression not including the height predictor. Genetic principal components were calculated from LD pruned HM3 SNPs (${r}_{{\rm{LD}}}^{2}\,$ < 0.1). We used height of siblings or parents as a predictor of height as well as various polygenic scores (PGSs) calculated as a weighted sum of height-increasing alleles. The direction and magnitude of these weights was determined by estimated SNP effects from our discovery GWAS meta-analyses. No calibration of tuning parameters in a validation was performed.

#### Between-family prediction

We analysed two classes of PGS. The first class is based on SNPs ascertained using GCTA-COJO. We applied GCTA-COJO to ancestry-specific and cross-ancestry GWAS meta-analyses using an ancestry-matched and an EUR LD reference, respectively. We compared PGSs based on SNPs ascertained at different significance thresholds: *P* < 5 × 10^−8^ (GWS: reported in the main text) and *P* < 5 × 10^−7^, *P* < 5 × 10^−6^ and *P* < 5 × 10^−5^. For all COJO-based PGS, we used estimated joint effects to calculate the PGS. The second class of PGS uses weights for all HM3 SNPs obtained from applying the SBayesC method^28^ to ancestry-specific and cross-ancestry GWAS meta-analyses with ancestry-matched and EUR-specific LD matrices, respectively. The SBayesC method is a Bayesian PGS-method implemented in the GCTB software (v.2.0), which uses the same prior as the LDpred method^61,62^. In brief, SBayesC models the distribution of joint effects of all SNPs using a two-component mixture distribution. The first component is a point-mass Dirac distribution on zero and the other component a Gaussian distribution (for each SNP) with mean 0 and a variance parameter to estimate. Full LD matrices (that is, not sparse) were calculated using GCTB across around 250 overlapping (50% overlap) blocks of around 8,000 SNPs (average size is around 20 Mb). These LD matrices were calculated using the same sets of genotypes used for COJO analyses (described above). We ran SBayesC in each block separately with 100,000 Monte Carlo Markov Chain iterations. In each run, we initialized the proportion of causal SNPs in a block at 0.0001 and the heritability explained by SNPs in the block at 0.001. Posterior SNP effects of SNPs present in two blocks were meta-analysed using inverse-variance meta-analysis.

Prediction accuracy was quantified in 61,095 unrelated individuals from three studies, including 33,001 participants of the UKB who were not included in our discovery GWAS (that is, 14,587 EUR; 9,257 SAS; 6,911 AFR and 2,246 EAS; Methods section ‘Samples used for prediction and estimation of variance explained’); 14,058 EUR participants from the Lifelines cohort study; and 8,238 HIS and 5,798 AFR participants from the PAGE study.

#### Within-family prediction

The prediction accuracy of sibling’s height was assessed in 17,942 unrelated sibling pairs from the UKB. Those pairs were determined by intersecting the list of UKB sibling pairs determined by Bycroft et al.^63^ with a list of genetically determined European ancestry participants from the UKB also described previously^3^. We then filtered the resulting list for SNP-based genetic relationship between members of different families to be smaller than 0.05. The prediction accuracy of parental height (each parent and their average) was assessed in 981 unrelated trios obtained as described above by crossing information from Bycroft et al.^63^ (calling of relatives) with that from Yengo et al.^3^ (calling of European ancestry participants). We quantified the within-family variance explained by PGS as the squared correlation of height difference between siblings with PGS difference between siblings. We describe in Supplementary Note 4 how familial information and PGS were combined to generate a single predictor.

### Samples used for prediction and estimation of variance explained

We quantified the accuracy of a PGS based on GWS SNPs as well as the variance explained by SNPs within GWS loci, in eight different datasets independent of our discovery GWAS meta-analyses. These datasets include two samples of EUR from the UKB (*n* = 14,587) and the Lifelines study (*n* = 14,058), two samples of AFR from the UKB (*n* = 6,911) and the PAGE study (*n* = 8,238), two samples of EAS (*n* = 2,246) from the UKB and the China Kadoorie Biobank (CKB; *n* = 47,693), one sample of SAS from the UKB (*n* = 9,257) and one sample of HIS from the PAGE study (*n* = 4,939). Analyses were adjusted for age, sex, 20 genotypic principal components and study-specific covariates (for example, recruitment centres). Genotypes of EUR UKB participants were imputed to the Haplotype Reference Consortium (HRC) and to a combined reference panel including haplotypes from the 1KG Project and the UK10K Project. To improve variant coverage in non-EUR participants of UKB, we re-imputed their genotypes to the 1KG reference panel, as described previously^38^. Lifelines samples were imputed to the HRC panel. PAGE and CKB were imputed to the 1KG reference panel. Standard quality control (${r}_{{\rm{INFO}}}^{2}$ > 0.3, *P*_HWE_ > 10^−6^ and MAC > 5) were applied to imputed genotypes in each dataset.

### Contribution of LD and MAF to the loss of prediction accuracy

We defined the EUR-to-AFR relative accuracy as the ratio of prediction accuracies from an AFR sample over that from a EUR sample. We used a previously published method^38^ to quantify the expectation of that relative accuracy under the assumption that causal variants and their effects are shared between EUR and AFR, whereas MAF and LD structures can differ. In brief, this method contrasts LD and MAF patterns within 100-kb windows around each GWS SNPs and uses them to predict the expected loss of accuracy. As previously described^38^, we used genotypes from 503 EUR and 661 AFR participants of the 1KGP as a reference sample to estimate ancestry-specific MAF and LD correlations between GWS SNPs and SNPs in their close vicinity, and defined candidate causal variants as any sequenced SNP with an ${r}_{{\rm{LD}}}^{2}$ > 0.45 with a GWS SNP within that 100-kb window. Standard errors were calculated using a delta-method approximation as previously described^38^.

### Down-sampled GWAS analyses

In addition to our EUR GWAS meta-analysis and our trans-ancestry meta-analysis (META_FE_), we re-analysed five down-sampled GWASs as shown in Table 2. These down-sampled GWASs include various iterations of previous efforts of the GIANT consortium and have a sample size varying between around 130,000 and 2.5 million (EUR participants from 23andMe). To ensure sufficient genomic coverage of HM3 SNPs we imputed GWAS summary statistics from Lango Allen et al.^19^, Wood et al.^20^ and Yengo et al.^3^. with ImpG-Summary (v.1.0.1)^64^ using haplotypes from 1KGP as a LD reference. GWAS summary statistics from Lango Allen et al. only contain *P* values (*P*), height-increasing alleles and per-SNP sample sizes (*N*). Therefore, we first calculated *Z*-scores (*Z*) from *P* values assuming that *Z*-scores are normally distributed, then derived SNP effects (*β*) and corresponding standard errors (s.e.) using linear regression theory as $\beta =Z/\sqrt{2{\rm{MAF}}\times (1-{\rm{MAF}})\times \left(N+{Z}^{2}\right)}$ and SE = *β*/*Z*. Imputed GWAS summary statistics from these three studies are made publicly available on the GIANT consortium website (see ‘URLs’ section). We next performed a COJO analysis of all down-sampled GWAS using genotypes of 348,501 unrelated EUR participants in the UKB as a LD reference panel, as for our META_FE_ and EUR GWAS meta-analysis.

### Gene prioritization using SMR

We used SMR to identify genes whose expression could mediate the effects of SNPs on height. SMR analyses were performed using the SMR software v.1.03. We used publicly available gene eQTLs identified from two large eQTL studies; namely, the GTEx^65^ v.8 and the eQTLgen studies (see ‘URLs’ section). To ensure that our SMR results robustly reflect causality or pleiotropic effects of height-associated SNPs on gene expression, we only report here significant SMR results (that is, *P* < 5 × 10^−8^), which do not pass the heterogeneity in dependent instrument (HEIDI) test (that is, *P* > 0.01; Methods). The significance threshold for the HEIDI test was chosen on the basis of recommendations from another study^66^.

### Selection of OMIM genes

To generate a list of genes that are known to underlie syndromes of abnormal skeletal growth, we queried the Online Mendelian Inheritance in Man database (OMIM; https://www.omim.org/). From July 2019 to August 2020, we performed queries using search terms of “short stature”, “tall stature”, “overgrowth”, “skeletal dysplasia” and “brachydactyly.” We then used the free text descriptions in OMIM to manually curate the resulting combined list of genes, as well as genes in our earlier list from Wood et al.^20^ and all genes listed as causing skeletal disease in an online endocrine textbook (https://www.endotext.org/, accessed September 2020). For short stature, we only included genes that underlie syndromes in which short stature was either consistent (less than −2 s.d. in the vast majority of patients with data recorded), or present in multiple families or sibships and accompanied by (a) more severe short stature (−3 s.d.), (b) presence of skeletal dysplasia (beyond poor bone quality/fractures); or (c) presence of brachydactyly, shortened digits, disproportionate short stature or limb shortening (not simply absence of specific bones). We removed genes underlying syndromes in which short stature was likely to be attributable to failure to thrive, specific metabolic disturbances, intestinal failure or enteropathy and/or very severe disease (for example, early lethality or severe neurological disease). For tall stature or overgrowth, we only included genes underlying syndromes in which tall stature was consistent (more than +2 s.d. in the vast majority of patients with data recorded) or present in multiple families or sibships and accompanied by either (a) more severe tall stature (>+3 s.d.) or (b) arachnodactyly. For brachydactyly, we required more than only fifth finger involvement, and that brachydactyly be either consistent (present in the vast majority of patients) or accompanied by consistent short stature or other skeletal dysplasias. For skeletal dysplasias, we only considered genes that underlie syndromes in which the skeletal dysplasia involved long bones or the spine and was accompanied by short stature, brachydactyly or limb or digit shortening. We also included all genes in a list we generated in Lango Allen et al.^19^, which was curated using similar criteria. The resulting list contained 536 genes, of which 462 (Supplementary Table 11) are autosomal on the basis of annotation from PLINK (https://www.cog-genomics.org/static/bin/plink/glist-hg19).

### URLs

GIANT consortium data files: https://portals.broadinstitute.org/collaboration/giant/index.php/GIANT_consortium_data_files. Analysis script for within- and across-ancestry meta-analysis: https://github.com/loic-yengo/ScriptsForYengo2022_HeightGWAS/blob/main/run-meta-analyses-within-ancestries.R and https://github.com/loic-yengo/ScriptsForYengo2022_HeightGWAS/blob/main/run-meta-analyses-across-ancestries.R. Analysis script for correction of winner’s curse: https://github.com/loic-yengo/ScriptsForYengo2022_HeightGWAS/blob/main/WC_correction.R. Genotypes from 1KG: https://ftp.1000genomes.ebi.ac.uk/vol1/ftp/release/20130502/. eQTL data for SMR: GTEx v.8: https://yanglab.westlake.edu.cn/data/SMR/GTEx_V8_cis_eqtl_summary.html; eQTLgen: https://www.eqtlgen.org/cis-eqtls.html. Annotation-weighted LD scores for stratified LD score regression analyses: https://alkesgroup.broadinstitute.org/LDSCORE/LDSCORE/. LDSC software: https://github.com/bulik/ldsc.

### Reporting summary

Further information on research design is available in the Nature Research Reporting Summary linked to this article.

## Online content

Any methods, additional references, Nature Research reporting summaries, source data, extended data, supplementary information, acknowledgements, peer review information; details of author contributions and competing interests; and statements of data and code availability are available at 10.1038/s41586-022-05275-y.

## Supplementary information

Supplementary Information This file contains Acknowledgements, Supplementary Figures 1–25, Supplementary Notes 1–6, and Supplementary References.

Reporting Summary

Supplementary Tables This file contains Supplementary Tables 1–16.

Peer Review File

## Data Availability

Summary statistics for ancestry-specific and multi-ancestry GWASs (excluding data from 23andMe) as well as SNP weights for polygenic scores derived in this study are made publicly available on the GIANT consortium website (see ‘URLs’ for GIANT consortium data files). GWAS summary statistics derived involving 23andMe participants will be made available to qualified researchers under an agreement with 23andMe that protects the privacy of participants. Application for data access can be submitted at https://research.23andme.com/dataset-access/. We used genotypes from various publicly available databases to estimate linkage disequilibrium correlations required for conditional analyses and genome-wide prediction analyses. These databases include the UK Biobank under project 12505 and the database of Genotypes and Phenotypes (dbGaP) under project 15096. Accession numbers for dbGaP datasets are phs000788.v2.p3.c1, phs000386, phs000557.v4.p1, phs000286.v5.p1, phs000613.v1.p2, phs000284.v2.p1, phs000283.v7.p3 and phs001395.v2.p1 cohorts. Details for each dbGaP dataset are given in the Methods. Source data are provided with this paper.

## Source data

Source Data Fig. 1
Source Data Fig. 2
Source Data Fig. 3
Source Data Fig. 4
Source Data Extended Data Fig. 2
Source Data Extended Data Fig. 3
Source Data Extended Data Fig. 4
Source Data Extended Data Fig. 5
Source Data Extended Data Fig. 6
Source Data Extended Data Fig. 7
Source Data Extended Data Fig. 8
Source Data Extended Data Fig. 9
Source Data Extended Data Fig. 10

## Code availability

We used publicly available software tools for all analyses. These software tools are listed in the main text and in the Methods. Source data are provided with this paper.
