# Supplementary material for: A saturated map of common genetic variants associated with human height
Source: Nature. 2022 Oct 12;610(7933):704–12. doi: 10.1038/s41586-022-05275-y (PMC9605867; doi:10.1038/s41586-022-05275-y)
Supplement: Supplementary file 2 — Reporting Summary [file 41586_2022_5275_MOESM2_ESM.pdf]

nature portfolio

Corresponding author(s): Dr Loic Yengo

Last updated by author(s): Jun 19, 2022

Reporting Summary

Nature Portfolio wishes to improve the reproducibility of the work that we publish. This form provides structure for consistency and transparency in reporting. For further information on Nature Portfolio policies, see our Editorial Policies and the Editorial Policy Checklist.

Statistics

For all statistical analyses, confirm that the following items are present in the figure legend, table legend, main text, or Methods section.

n/a	Confirmed	
☐	☒	The exact sample size (*n*) for each experimental group/condition, given as a discrete number and unit of measurement
☒	☐	A statement on whether measurements were taken from distinct samples or whether the same sample was measured repeatedly
☐	☒	The statistical test(s) used AND whether they are one- or two-sided<br>*Only common tests should be described solely by name; describe more complex techniques in the Methods section.*
☐	☒	A description of all covariates tested
☐	☒	A description of any assumptions or corrections, such as tests of normality and adjustment for multiple comparisons
☐	☒	A full description of the statistical parameters including central tendency (e.g. means) or other basic estimates (e.g. regression coefficient) AND variation (e.g. standard deviation) or associated estimates of uncertainty (e.g. confidence intervals)
☐	☒	For null hypothesis testing, the test statistic (e.g. *F*, *t*, *r*) with confidence intervals, effect sizes, degrees of freedom and *P* value noted<br>*Give P values as exact values whenever suitable.*
☐	☒	For Bayesian analysis, information on the choice of priors and Markov chain Monte Carlo settings
☒	☐	For hierarchical and complex designs, identification of the appropriate level for tests and full reporting of outcomes
☒	☐	Estimates of effect sizes (e.g. Cohen's *d*, Pearson's *r*), indicating how they were calculated

Our web collection on statistics for biologists contains articles on many of the points above.

Software and code

Policy information about availability of computer code

Data collection	Genotyping arrays and calling algorithms are listed for all cohorts in ST2.
Data analysis	Statistical analyses were performed with R version v4.21 (for simulations, visualization, regression and prediction analyses), PLINK v1.9 for standard quality control and GWAS analyses, GCTA v1.93 for estimating SNP-based heritability and for conditional analyses (COJO algorithm), GCTB v2.0 for running the SBayesC model used in prediction analyses, BOLT-LMM v2 and rvtest for mixed-model based GWAS analyses (versions listed in ST2), KING v2.2.5 for identifying relatives in certain cohorts, RAREMETAL v4.15.1. for running within-ancestry GWAS meta-analyses, LDSC v1.0.0 for LD score regression analyses, ImpG-Summary v1.0.1 for imputing GWAS summary statistics, SMR v1.03 for eQTL-based Mendelian Randomization analyses. Additional scripts used for analyses are listed in the Code Availability Statement.

For manuscripts utilizing custom algorithms or software that are central to the research but not yet described in published literature, software must be made available to editors and reviewers. We strongly encourage code deposition in a community repository (e.g. GitHub). See the Nature Portfolio guidelines for submitting code & software for further information.

Data

Policy information about availability of data

All manuscripts must include a data availability statement. This statement should provide the following information, where applicable:

- Accession codes, unique identifiers, or web links for publicly available datasets
- A description of any restrictions on data availability
- For clinical datasets or third party data, please ensure that the statement adheres to our policy

Summary statistics for ancestry-specific and multi-ancestry GWAS (excluding data from 23andMe) as well as SNP weights for polygenic scores derived in this study are made publicly available at https://portals.broadinstitute.org/collaboration/giant/index.php/GIANT_consortium_data_files. GWAS summary statistics derived

involving 23andMe participants will be made available to qualified researchers under an agreement with 23andMe that protects the privacy of participants. Application for data access can be submitted at https://research.23andme.com/dataset-access/. We used genotypes from various publicly available databases to estimate linkage disequilibrium correlations required for conditional analyses and genome-wide prediction analyses. These databases include the UK Biobank under project 12505 and the database of Genotypes and Phenotypes (dbGaP) under project 15096. Accession numbers for dbGaP datasets are phs000788.v2.p3.c1, phs000386, phs000557.v4.p1, phs000286.v5.p1, phs000613.v1.p2, phs000284.v2.p1, phs000283.v7.p3 and phs001395.v2.p1 cohorts. Details for each dbGaP dataset are given in the Methods section.

Field-specific reporting

Please select the one below that is the best fit for your research. If you are not sure, read the appropriate sections before making your selection.

☒ Life sciences ☐ Behavioural & social sciences ☐ Ecological, evolutionary & environmental sciences

For a reference copy of the document with all sections, see nature.com/documents/nr-reporting-summary-flat.pdf

Life sciences study design

All studies must disclose on these points even when the disclosure is negative.

Sample size	We meta-analyzed GWAS data from 281 cohorts, with the aim to reach the largest possible sample size (here, N=5.4 million). Our sample size is larger than any previous GWAS of height, thereby expected to deliver additional associations.
Data exclusions	Samples were excluded based on heterozygosity, low call rate, relatedness relative to other participants of the same cohort. Details for each cohort are given in ST2. We analyzed SNPs with a minor allele frequency >1% in at least one of five ancestry groups. Other quality control exclusions are listed in ST2.
Replication	We replicated associations detected in our study in N=49,160 individuals in the Estonian Biobank. Quantification of variance explained by height-associated SNPs and prediction accuracy was performed in 61,095 individuals independent of our discovery dataset from four cohorts (hold-out sample from the UK Biobank, the Lifelines Study, the Chinese Kadoorie Biobank and the PAGE Study).
Randomization	N/A. Our study is observational and used data from all available participants. No intervention was implemented in any of the study participants, therefore randomization was not required.
Blinding	N/A. No intervention was implemented in any of the study participants, therefore blinding was not required.

Reporting for specific materials, systems and methods

We require information from authors about some types of materials, experimental systems and methods used in many studies. Here, indicate whether each material, system or method listed is relevant to your study. If you are not sure if a list item applies to your research, read the appropriate section before selecting a response.

Materials & experimental systems

n/a	Involved in the study	
☒	☐	Antibodies
☒	☐	Eukaryotic cell lines
☒	☐	Palaeontology and archaeology
☒	☐	Animals and other organisms
☐	☒	Human research participants
☒	☐	Clinical data
☒	☐	Dual use research of concern

Methods

n/a	Involved in the study	
☒	☐	ChIP-seq
☒	☐	Flow cytometry
☒	☐	MRI-based neuroimaging

Human research participants

Policy information about studies involving human research participants

Population characteristics	The mean/standard deviation/ minimum and maximum values for height and age is given separately for males and females of each cohort in ST3.
Recruitment	Study participants were recruited under various designs including population based, prospective studies, birth cohorts, family-based, hospital-based, (nested) case-controls, case-cohort, clinical trials. Designs for each cohort is listed in ST1.
Ethics oversight	Written informed consent was obtained from every participant in each study, and the study was approved by relevant ethics committees for each cohort. We provide a list of Institutional Review Boards in ST1.

Note that full information on the approval of the study protocol must also be provided in the manuscript.
